# Anticodon Engineered Transfer RNA (tRNA^SUAG^) Inhibits Hepatitis B Virus Replication by Promoting the Degradation of Core Protein

**DOI:** 10.1002/advs.202503534

**Published:** 2025-09-12

**Authors:** Xingwen Yang, Huiying Sun, Ziheng Luo, Qinxin Zhang, Yarong Song, Jie Li, Xiaoyun Liu, Jie Wang

**Affiliations:** ^1^ Department of Microbiology & Infectious Disease Center School of Basic Medical Sciences Peking University Health Science Center 38 Xueyuan Road, Haidian District Beijing 100191 China; ^2^ NHC Key Laboratory of Medical Immunology Peking University Beijing 100191 China

**Keywords:** anticodon engineered transfer RNA, tRNA^SUAG^, hepatitis B virus, core protein, ACE‐tRNA‐gRNA tandem array

## Abstract

Current antiviral drugs targeting the hepatitis B virus (HBV) still face challenges in achieving a functional cure for chronic hepatitis B. Therefore, it is necessary to explore new therapeutic approaches and combined therapy strategies. Anticodon engineered transfer RNA (ACE‐tRNA) has been widely applied in the treatment of genetic diseases caused by nonsense mutations. However, its role in antiviral therapy has not been reported yet. In this study, ACE‐tRNAs are designed to target the highly conserved stop codon (UAG) of HBV core protein (HBc). All designed ACE‐tRNAs can read through the stop codon of HBc. Among them, tRNA^SUAG^ might promote HBc phosphorylation by introducing a phosphorylatable serine into the C‐terminal domain of HBc, thereby potentially reducing HBc levels and inhibiting HBV replication by promoting the degradation of HBc through the ubiquitin‐proteasome pathway. In addition, ACE‐tRNA and CRISPR/Cas9 technologies can be effectively integrated through a tRNA^SUAG^‐gRNA tandem array and achieve the combined inhibition of HBV replication. This study innovatively applied ACE‐tRNA to promote HBc degradation by introducing an amino acid that may be post‐translationally modified and subsequently inhibit HBV replication. In addition, this study presents a promising therapeutic strategy for promoting the clearance of HBV infection by integrating ACE‐tRNA and CRISPR/Cas9 technology.

## Graphical abstract

This study employed tRNA^SUAG^, an anticodon‐engineered tRNA (ACE‐tRNA), to introduce a phosphorylatable serine into the C‐terminal domain of HBc by reading through its stop codon, thereby potentially promoting the ubiquitin‐proteasome degradation of HBc, and subsequently inhibiting HBV replication. Through a tRNA^SUAG^‐gHBV1‐tRNA^SUAG^‐gHBV2‐tRNA^SUAG^ tandem array, a combined inhibition of HBV replication can be achieved by integrating ACE‐tRNA and CRISPR/Cas9 technology.

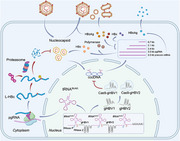

## 1 Introduction

Hepatitis B virus (HBV) infection remains a major global health concern. According to the World Health Organization, ≈254 million people are living with chronic HBV infection, and ≈1.1 million people die from HBV‐related end‐stage liver disease, including cirrhosis and hepatocellular carcinoma.^[^ ^1^ ^]^ Current anti‐HBV drugs, including nucleos(t)ide analogues and pegylated‐interferon α, still face challenges in achieving a functional cure for chronic hepatitis B.^[^ ^2^, ^3^, ^4^ ^]^ Therefore, the development of new therapeutic approaches and combination therapy strategies is necessary to achieve the global goal of eliminating viral hepatitis by 2030.

The HBV core protein (HBc) plays a crucial role in HBV replication and has cytopathic effects associated with severe hepatitis, making it a promising therapeutic target for HBV infection and HBV infection related liver diseases.^[^ ^5^, ^6^ ^]^ Once HBV pre‐genomic RNA (pgRNA) is transcribed from covalently closed circular DNA (cccDNA), HBc will be translated from pgRNA, and some HBc will be assembled into nucleocapsids. Mature nucleocapsids containing relaxed circular DNA (rcDNA) can be secreted outside hepatocytes to form viral particles or transported to the nucleus to supplement the cccDNA pool.^[^ ^7^ ^]^ At present, capsid assembly modulators (CAMs) targeting the assembly process of HBc have been widely explored in clinical trials.^[^ ^8^ ^]^ Although CAMs can effectively inhibit HBV replication, they have certain limitations. For example, the emergence of HBV variants that resist CAMs can lead to virological breakthroughs, and certain CAMs‐induced liver damage can result in elevated levels of alanine transaminase (ALT).^[^ ^9^, ^10^, ^11^ ^]^ Therefore, it is necessary to develop new approaches targeting HBc with low toxicity and drug resistance.

Anticodon engineered transfer RNA (ACE‐tRNA) can specifically recognize a designated stop codon (UAA‐ochre codon, UAG‐amber codon, or UGA‐opal codon) by modifying the anticodon loop of natural tRNA.^[^ ^12^ ^]^ ACE‐tRNA can efficiently read through premature termination (stop) codons (PTC) within the open reading frame (ORF) of functional proteins and restore the generation of full‐length proteins.^[^ ^13^ ^]^ However, the natural stop codons can hardly be read through by ACE‐tRNA.^[^ ^14^, ^15^ ^]^ Therefore, ACE‐tRNA holds promising potential for treating various genetic disorders caused by nonsense mutations, such as β‐thalassemia, cystic fibrosis, and Duchenne muscular dystrophy, and can be delivered by adeno‐associated virus (AAV) or lipid nanoparticle.^[^ ^14^, ^15^, ^16^, ^17^, ^18^ ^]^

HBV genome is a partially double‐stranded DNA of ≈3.2 kb and contains four overlapping ORFs: C, P, S, and X.^[^ ^5^ ^]^ HBc in the C ORF and HBV DNA polymerase in the P ORF are both translated from pgRNA. Due to the partial overlap between the C and P ORFs, and the presence of another in‐frame stop codon (UAA) located at the 6th codon behind the normal stop codon (UAG) of C ORF, the normal stop codon (UAG) of C ORF resembles PTC in some aspects. Therefore, it is possible for ACE‐tRNA to read through the normal stop codon of C ORF, which may be a potential approach for treating HBV infection. Although ACE‐tRNA has been widely used for treating various genetic disorders by reading through PTC, its role in antiviral therapy has not been reported yet. Based on the secondary structure of RNA chains rather than the specific nucleotide sequences, the 5’ leader sequence and 3’ trailer sequence of tRNA are cleaved by RNase P and RNase Z during tRNA maturation, respectively.^[^ ^19^ ^]^ This unique characteristic enables tRNA to serve as a scaffold for tandem expression of multiplex non‐coding RNAs.^[^ ^20^ ^]^ For the clustered regularly interspaced short palindromic repeats (CRISPR)/CRISPR‐associated nuclease 9 (Cas9) system, the tRNA‐guide RNA (gRNA) tandem array has successfully achieved tandem expression of multiplex gRNAs in *Oryza sativa*,^[^ ^20^ ^]^ *Drosophila melanogaster*,^[^ ^21^ ^]^ and *Saccharomyces cerevisiae*.^[^ ^22^ ^]^ However, research on tRNA‐gRNA tandem arrays in mammalian cells is limited.^[^ ^23^ ^]^

In this study, we first explored whether ACE‐tRNA can read through the stop codon of C ORF. Then, the effect of ACE‐tRNA on HBV replication and its underlying mechanisms was explored. In addition, we explored whether ACE‐tRNA and CRISPR/Cas9 technology can be effectively integrated through ACE‐tRNA‐gRNA tandem arrays to achieve the combined inhibition of HBV replication.

## 2 Results

### 2.1 ACE‐tRNA Can Read Through the Stop Codon of C ORF

**Figure 1:**
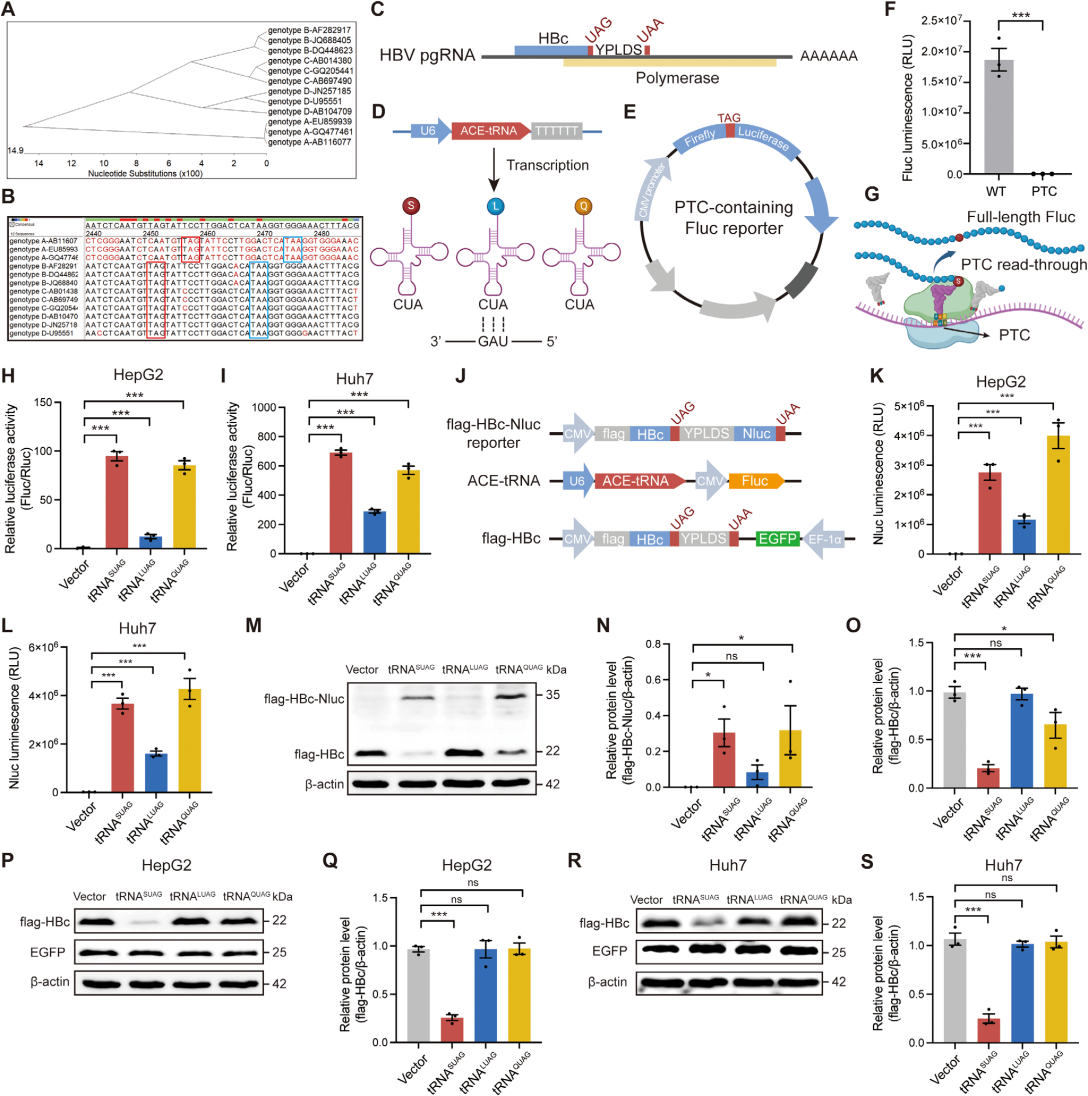
Analyzing the readthrough efficiency of ACE‐tRNAs targeting UAG. A) Phylogenetic tree of genotypes A‐D HBV genome reference sequences. B) Homology analysis of stop codon in C ORF. C) The diagram of genotype C HBV pgRNA encoding HBc and polymerase. D) The diagram of the ACE‐tRNA expression cassette. S: serine, L: leucine, Q: glutamine. E) The diagram of the PTC‐containing Fluc reporter. F) The wild‐type or PTC‐containing Fluc reporter was transfected into HEK293T cells, and the activity of Fluc was detected at 48 h post‐transfection. G) The diagram illustrates the restoration of full‐length Fluc mediated by ACE‐tRNAs. The PTC‐containing Fluc reporter, Renilla luciferase (Rluc) expression plasmid (pRL‐TK) and each ACE‐tRNA expression plasmid were co‐transfected into H) HepG2 and I) Huh7 cells, and the relative luciferase activity (Fluc/Rluc) was detected at 48 h post‐transfection. J) The diagrams of genotype C HBc‐Nluc reporter, ACE‐tRNA, and Fluc expression cassette, as well as flag‐HBc and EGFP expression cassette. The HBc‐Nluc reporter and each ACE‐tRNA expression plasmid were co‐transfected into K) HepG2 and L) Huh7 cells. The activity of Nluc was detected at 48 h post‐transfection. M) The levels of flag‐HBc‐Nluc fusion protein and flag‐HBc in HepG2 cells were detected by Western blotting. The relative protein levels of N) flag‐HBc‐Nluc fusion protein and O) flag‐HBc protein were analyzed by ImageJ. The pCDH‐flag‐HBc and each ACE‐tRNA expression plasmid were co‐transfected into HepG2 cells. P) The levels of flag‐HBc and EGFP were detected by Western blotting. Q) The relative level of HBc protein was analyzed by ImageJ. The pCDH‐flag‐HBc and each ACE‐tRNA expression plasmid were co‐transfected into Huh7 cells. R) The levels of flag‐HBc and EGFP were detected by Western blotting. S) The relative level of HBc protein was analyzed by ImageJ. *p < 0.05, ***p < 0.001, ns indicated no significance, two‐tailed *t*‐test.

Through sequence analysis, we found that the normal stop codon of C ORF is UAG and highly conserved (**Figure** **1**A,B). UAG in the C ORF is located at nt2450‐2452 in the genomes of genotypes B‐D HBV but at nt2456‐2458 in the genome of genotype A HBV, due to the insertion of six nucleotides (GGACCG) at nt2356‐2361 in the genome of genotype A HBV (Figure 1B). The stop codon is UGA in the P ORF, UAA or UGA in the S ORF, and UAA in the X ORF (Figure S1A–C, Supporting Information). Therefore, UAG is the specific stop codon of C ORF. As shown in Figure 1B,C, HBc in the C ORF and polymerase in the P ORF are both translated from pgRNA. P ORF is located behind the C ORF and partially overlaps with the C ORF. In addition, another highly conserved in‐frame stop codon UAA is located behind UAG, and there are five codons between the two stop codons, making the normal stop codon of C ORF like PTC in some aspects. Therefore, it is possible for ACE‐tRNA to read through the UAG of C ORF.

Based on Lueck's high‐throughput screening on the readthrough efficiency of ACE‐tRNAs,^[^ ^14^ ^]^ the top three ACE‐tRNAs targeting UAG were selected and named tRNA^SUAG^, tRNA^LUAG^, and tRNA^QUAG^, which originated from the natural tRNA carrying serine (S), leucine (L), and glutamine (Q), respectively (Figure 1D). To assess the readthrough efficiency of the three ACE‐tRNAs, a firefly luciferase (Fluc) reporter containing a PTC was constructed by inserting TAG upstream of the acyl‐activating enzyme consensus motif of Fluc (Figure 1E). As shown in Figure 1F, PTC effectively impeded the catalytic activity of Fluc by prematurely terminating its translation. Once ACE‐tRNA successfully reads through PTC, full‐length Fluc will be translated (Figure 1G). The results showed that three ACE‐tRNAs could read through PTC in the Fluc reporter, and the readthrough efficiency was highest for tRNA^SUAG^ (Figure 1H,I).

To further explore whether ACE‐tRNA can read through the UAG of C ORF, an HBc‐Nano luciferase (Nluc) reporter was constructed by inserting the Nluc ORF behind the UAG of C ORF, and ACE‐tRNA expression plasmids capable of simultaneously expressing Fluc through the CMV promoter were constructed (Figure 1J). Once ACE‐tRNA reads through the UAG of C ORF, Nluc will be translated. The results showed that three ACE‐tRNAs could read through the UAG of C ORF, and the readthrough efficiency appeared to be the highest for tRNA^QUAG^ after excluding the differences in transfection efficiency reflected by Fluc activity (Figure 1K,L; Figure S1D,E, Supporting Information). Although the level of flag‐HBc‐Nluc fusion protein induced by tRNA^QUAG^ was highest and consistent with the activity of Nluc, the level of flag‐HBc protein induced by tRNA^SUAG^ was lowest (Figure 1M–O). To further confirm the effects of ACE‐tRNAs on HBc level, a flag‐HBc expression plasmid capable of simultaneously expressing enhanced green fluorescent protein (EGFP) through the EF‐1α promoter was constructed (Figure 1J). The results showed that tRNA^SUAG^ significantly reduced the level of HBc protein after excluding the differences in transfection efficiency reflected by Fluc activity and EGFP level, but not the other two ACE‐tRNAs (Figure 1P–S, Figure S1F,G, Supporting Information). Moreover, tRNA^SUAG^ reduced the level of HBc protein in a dose‐dependent manner (Figure S1H, Supporting Information). These results suggested that ACE‐tRNAs can read through the stop codon of C ORF with different efficiencies, whereas only tRNA^SUAG^ can reduce the level of HBc protein.

### 2.2 tRNA^SUAG^ Inhibits HBV Replication by Reducing HBc Level

**Figure 2:**
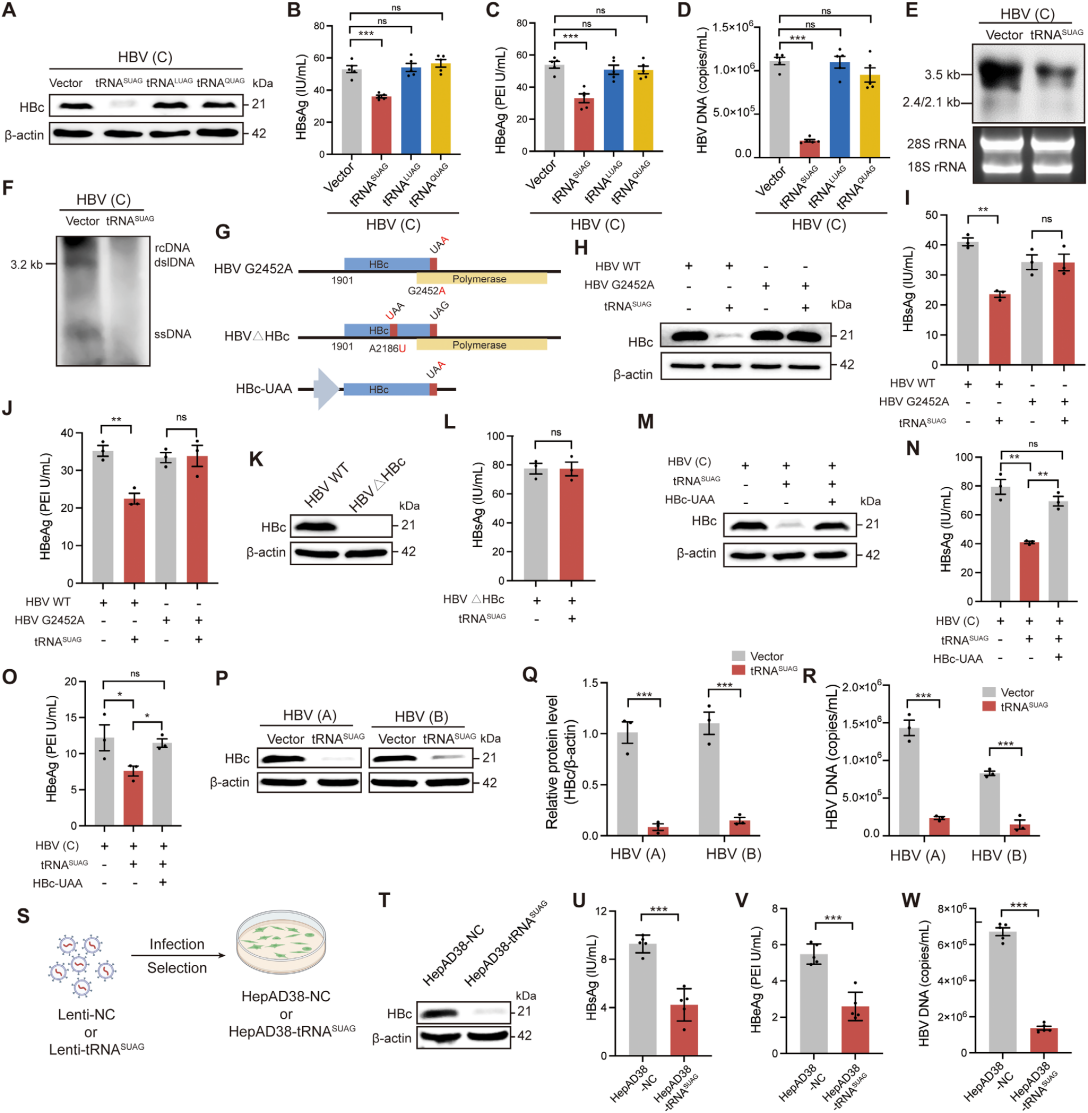
The effects of ACE‐tRNAs on genotypes A‐D HBV replication. Genotype C 1.3×HBV plasmid and each ACE‐tRNA expression plasmid were co‐transfected into HepG2 cells. A) The level of HBc protein was detected by Western blotting. The levels of B) HBsAg and C) HBeAg in the culture supernatants were detected by chemiluminescence immunoassays. D) The level of HBV DNA in the culture supernatants was detected by qPCR. Genotype C 1.3×HBV plasmid and tRNA^SUAG^ expression plasmid were co‐transfected into HepG2 cells. E) The level of intracellular HBV RNA was detected by Northern blotting. F) The level of intracellular HBV DNA was detected by Southern blotting. G) The diagram of the genotype C 1.3×HBV plasmid with HBc stop codon mutation (HBV G2452A), the genotype C 1.3×HBV plasmid with HBc nonsense mutation (HBV△HBc), and the HBc expression plasmid with a stop codon UAA (HBc‐UAA). Genotype C 1.3×HBV or 1.3×HBV G2452A plasmid and tRNA^SUAG^ expression plasmid or vector control were co‐transfected into HepG2 cells. H) The level of HBc protein was detected by Western blotting. The levels of I) HBsAg and J) HBeAg in the culture supernatants were detected by chemiluminescence immunoassay. K) Genotype C 1.3×HBV or 1.3×HBV△HBc plasmid was transfected into HepG2 cells, and the level of HBc protein was detected by Western blotting. L) Genotype C 1.3×HBV△HBc plasmid and tRNA^SUAG^ expression plasmid or vector control were co‐transfected into HepG2 cells, and the level of HBsAg in the culture supernatants was detected by chemiluminescence immunoassay. Genotype C 1.3×HBV plasmid, tRNA^SUAG^ expression plasmid or vector control, and HBc‐UAA expression plasmid or vector control were co‐transfected into HepG2 cells. M) The level of HBc protein was detected by Western blotting. The levels of N) HBsAg and O) HBeAg in the culture supernatants were detected by chemiluminescence immunoassays. The tRNA^SUAG^ expression plasmid or vector control and genotype A or B 1.3×HBV plasmid were co‐transfected into HepG2 cells. P) The level of HBc protein was detected by Western blotting. Q) The relative level of HBc protein was analyzed by ImageJ. R) The level of HBV DNA in the culture supernatants was detected by qPCR. S) The diagram on the construction of HepAD38‐NC and HepAD38‐tRNA^SUAG^ cells. HepAD38‐NC and HepAD38‐tRNA^SUAG^ cells were cultured without doxycycline (Dox) for a week and were seeded into a 6‐well plate. T) The level of HBc protein was detected by Western blotting. The levels of U) HBsAg and V) HBeAg in the culture supernatants were detected by chemiluminescence immunoassays. W) The level of HBV DNA in the culture supernatants was detected by qPCR. *p < 0.05, **p < 0.01, ***p < 0.001, ns indicated no significance, two‐tailed *t*‐test.

Next, we found that after excluding the transfection efficiency differences reflected by Fluc activity (Figure S2A, Supporting Information), only tRNA^SUAG^ significantly inhibited genotype C HBV replication by reducing the levels of HBc, hepatitis B surface antigen (HBsAg), hepatitis B e antigen (HBeAg), and HBV DNA (**Figure** **2**A–D). After evaluating the transfection efficiency of tRNA^SUAG^ using tRNA^SUAG^‐specific reverse transcription (RT)‐quantitative polymerase chain reaction (qPCR) analysis (Figure S2B, Supporting Information), we further confirmed that tRNA^SUAG^ significantly inhibited HBV replication through Northern blotting and Southern blotting analyses (Figure 2E,F). To confirm whether tRNA^SUAG^ inhibits HBV replication by reading through the stop codon UAG of HBc, we constructed a genotype C 1.3×HBV G2452A mutant plasmid, in which the stop codon of HBc was mutated from UAG to UAA (Figure 2G). As shown in Figure 2H–J, the decrease in HBc protein levels mediated by tRNA^SUAG^ and its inhibitory effect on HBV replication disappeared when the stop codon of HBc was mutated from UAG to UAA, suggesting that tRNA^SUAG^ reduced HBc protein levels and inhibited HBV replication by reading through the stop codon UAG of HBc. Further, we found that the nucleotide immediately following the stop codon UAG of HBc is U and highly conserved in the C ORF (Figure 1B). Considering that the nucleotide immediately following the stop codon makes a great influence on termination efficiency,^[^ ^15^ ^]^ we constructed a Fluc‐HBc context‐Nluc reporter, which contains the stop codon UAG of HBc, along with nine nucleotides upstream and nine nucleotides downstream. Apart from the wild‐type context, the nucleotide immediately following the stop codon was also mutated to A, G, and C, respectively (Figure S2C, Supporting Information). The readthrough efficiencies of the HBc stop codon UAG with different contexts were detected, and the result showed that UAGU was the most prone to be read through by tRNA^SUAG^ compared to UAGA, UAGG, and UAGC (Figure S2D, Supporting Information). To explore whether tRNA^SUAG^ inhibited HBV replication by reducing HBc level, we constructed a genotype C 1.3×HBV plasmid with HBc nonsense mutation (HBV△HBc) by introducing a premature stop codon UAA behind the C2 initiation codon (Figure 2G), according to the potential HBc translation initiation sites.^[^ ^24^ ^]^ The effect of tRNA^SUAG^ in inhibiting HBV replication disappeared when HBc expression was blocked (Figure 2K,L). We also constructed an HBc expression plasmid with a UAA stop codon (HBc‐UAA) (Figure 2G), and found that overexpression of HBc‐UAA rescued the inhibitory effect of tRNA^SUAG^ on HBV replication (Figure 2M–O). These results suggested that tRNA^SUAG^ inhibited HBV replication by reducing HBc level. In addition, tRNA^SUAG^ also significantly inhibited genotype A or B HBV replication by reducing HBc level (Figure 2P‐R).

Next, using a lentiviral expression system, we established HepAD38‐tRNA^SUAG^ cells that stably expressed tRNA^SUAG^ in HepAD38 cells, conditionally producing genotype D HBV (Figure 2S). The expression of tRNA^SUAG^ in HepAD38‐tRNA^SUAG^ cells was confirmed by HBc‐Nluc reporter (Figure S2E, Supporting Information). Consistent with other genotypes, tRNA^SUAG^ also significantly inhibited genotype D HBV replication by reducing HBc level (Figure 2T–W), without causing cytotoxicity (Figure S2F, Supporting Information).

### 2.3 tRNA^SUAG^ Inhibits HBV Replication in HBV‐Infected Cell Model

**Figure 3:**
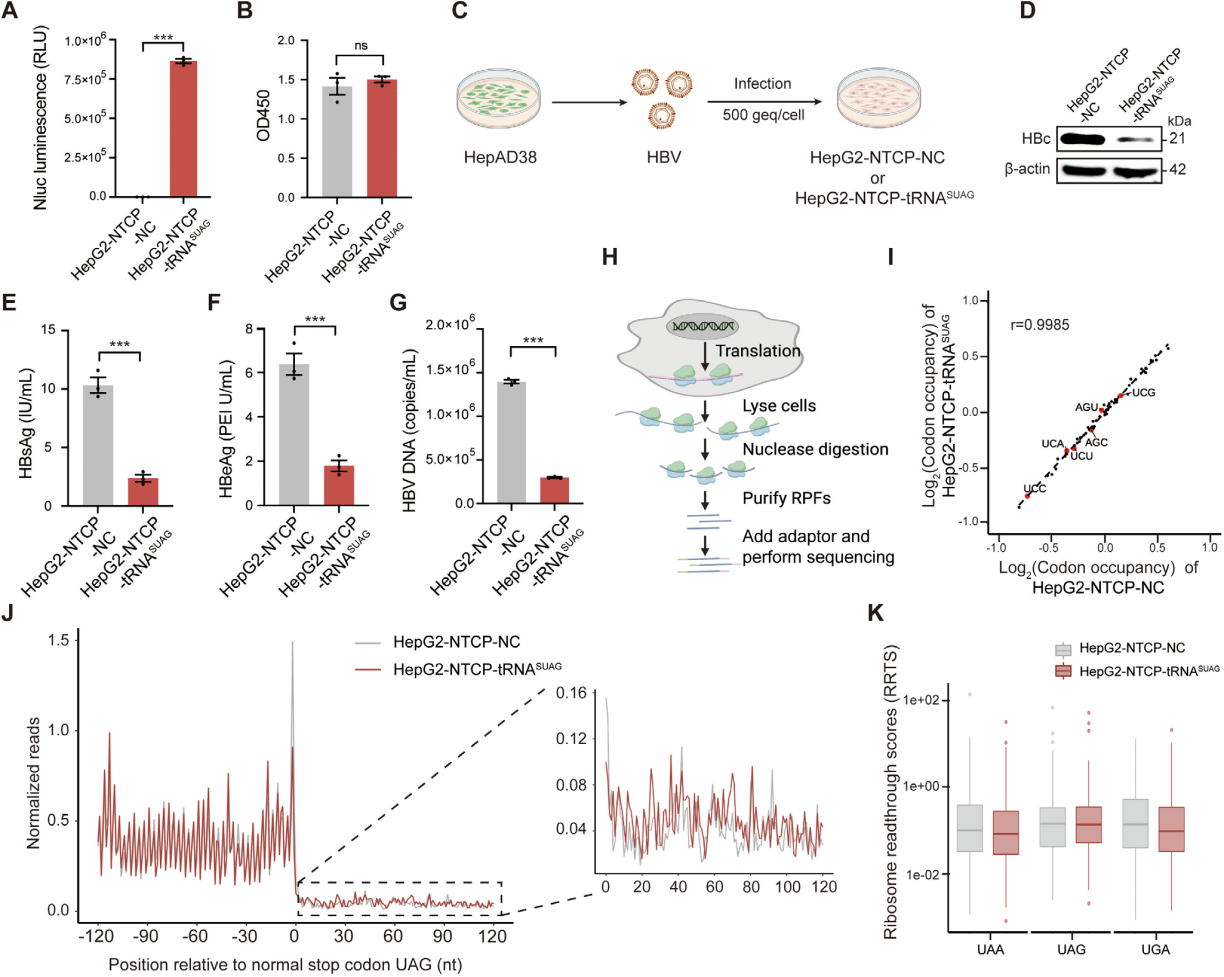
The effect of tRNA^SUAG^ on HBV replication under HBV infection. A) HBc‐Nluc reporter was transfected into HepG2‐NTCP‐NC and HepG2‐NTCP‐tRNA^SUAG^ cells, and the activity of Nluc was detected at 48 h post‐transfection. B) HepG2‐NTCP‐NC and HepG2‐NTCP‐tRNA^SUAG^ cells were seeded into a 96‐well plate, and CCK‐8 assays were performed after 48 h. C) The diagram on HBV infection (500 geq/cell) of HepG2‐NTCP‐NC and HepG2‐NTCP‐tRNA^SUAG^ cells. D) At 7 days post‐infection, the level of HBc protein was detected by Western blotting. The levels of E) HBsAg and F) HBeAg in the culture supernatants were detected by chemiluminescence immunoassays. G) The level of HBV DNA in the culture supernatants was detected by qPCR. H) The diagram of Ribo‐seq analysis. RPFs: ribosome‐protected mRNA fragments. Ribo‐seq analyses were performed in HepG2‐NTCP‐NC and HepG2‐NTCP‐tRNA^SUAG^ cells. I) The scatter plot showed the codon occupancy by exhibiting the RPF densities at each codon, and six serine codons were highlighted in red. J) The metagene plot showed the normalized RPFs relative to the distance from the normal stop codon UAG at position 0. K) The box plot of RRTS values calculated for transcripts harboring normal stop codons UAA, UAG, and UGA. ***p < 0.001, ns indicated no significance, two‐tailed *t*‐test.

To explore the effect of tRNA^SUAG^ on HBV replication under HBV infection, we constructed HepG2‐NTCP‐tRNA^SUAG^ cells stably expressing tRNA^SUAG^ in HepG2‐NTCP cells and their negative control HepG2‐NTCP‐NC cells. The expression of tRNA^SUAG^ in HepG2‐NTCP‐tRNA^SUAG^ cells was confirmed by HBc‐Nluc reporter (**Figure** **3**A). Meanwhile, no cytotoxicity was found in HepG2‐NTCP‐tRNA^SUAG^ cells (Figure 3B). As shown in Figure 3C, HepG2‐NTCP‐NC and HepG2‐NTCP‐tRNA^SUAG^ cells were infected with HBV produced by HepAD38 cells at 500 genome equivalents per cell (500 geq/cell), and the effect of tRNA^SUAG^ on HBV replication was detected at 7 days post‐infection. The results showed that tRNA^SUAG^ also significantly inhibited HBV replication by reducing HBc level in the HBV‐infected HepG2‐NTCP‐tRNA^SUAG^ cells (Figure 3D–G).

To exclude the possibility of interference caused by non‐specific readthrough for the normal stop codon UAG induced by tRNA^SUAG^ in human genes, we performed ribosome profiling (Ribo‐seq) analyses in HepG2‐NTCP‐NC and HepG2‐NTCP‐tRNA^SUAG^ cells (Figure 3H). As shown in Figure 3I, the occupancy of each serine codon was not significantly affected by tRNA^SUAG^, indicating that tRNA^SUAG^ did not significantly affect tRNA homeostasis. The abundance of ribosome‐protected mRNA fragments (RPFs) mapped to 3’ untranslated regions (UTRs) of mRNA with the stop codon UAG was comparable between the two cells (Figure 3J), suggesting that the non‐specific readthrough rate for the natural stop codon UAG induced by tRNA^SUAG^ was comparable to the physiological readthrough rate induced by near‐cognate tRNAs in human genes. Further, the ribosome readthrough score (RRTS) values of all mRNAs with stop codons UAA, UAG, or UGA were calculated. The result showed that tRNA^SUAG^ did not cause significant readthrough at all natural stop codons (Figure 3K). Moreover, quantitative proteomics analysis also confirmed that tRNA^SUAG^ was difficult to read through the normal stop codon UAG of human genes (Figure S3A,B, Supporting Information), and there was no enrichment of pathways with potential cytotoxicity (Figure S3C–E, Supporting Information).

### 2.4 tRNA^SUAG^ Inhibits HBV Replication In Vivo

**Figure 4:**
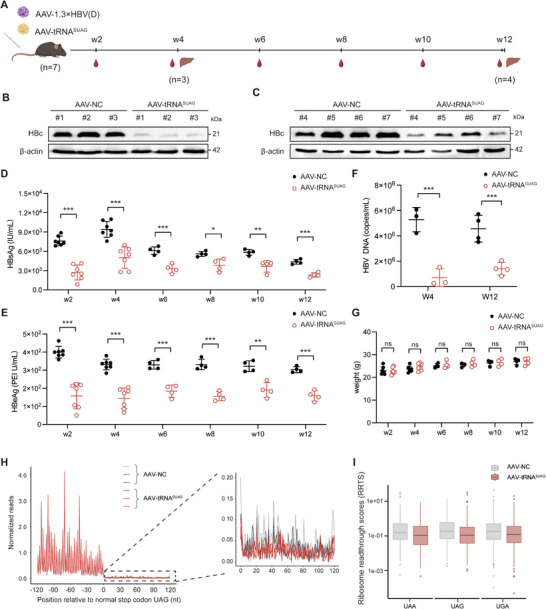
The effect of tRNA^SUAG^ on HBV replication in vivo. A) The workflow on the transduction of AAV8‐1.3×HBV (genotype D) and AAV8‐tRNA^SUAG^ in C57BL/6J mice. B) At 4 weeks and C) 12 weeks post‐injection, the level of HBc protein in the liver tissues of mice was detected by Western blotting. The levels of D) HBsAg and E) HBeAg in the serum were detected by chemiluminescence immunoassays every two weeks. F) The level of HBV DNA in the serum was detected by qPCR at 4 weeks and 12 weeks postinjection. G) The body weight of mice was measured every two weeks. H) Ribo‐seq analyses were performed for the liver tissues of three mice in each group, and the metagene plot showed normalized RPFs relative to the distance from the normal stop codon UAG at position 0. I) The box plot of RRTS values calculated for transcripts harboring normal stop codons UAA, UAG, and UGA. *p < 0.05, **p < 0.01, ***p < 0.001, ns indicated no significance, two‐tailed *t*‐test.

To explore the effect of tRNA^SUAG^ on HBV replication in vivo, AAV serotype 8 (AAV8) carrying tRNA^SUAG^ and AAV8‐1.3×HBV (genotype D) were packaged and simultaneously injected into the tail vein of C57BL/6J mice to avoid immune interference. As shown in **Figure** **4**A, there were seven mice in each group, three mice in each group were sacrificed at 4 weeks post‐injection, and the other four mice in each group were sacrificed at 12 weeks post‐injection. The results showed that tRNA^SUAG^ significantly inhibited HBV replication by continuously reducing the levels of HBc, HBsAg, HBeAg, and HBV DNA in mice, indicating that tRNA^SUAG^ maintained functional integrity over time (Figure 4B–F). Consistently, the expression levels of tRNA^SUAG^ in the liver tissues of mice were confirmed to be stable up to 12 weeks postinjection (Figure S4A, Supporting Information). There was no significant difference in body weight between the two groups of mice (Figure 4G), and no obvious inflammation or damage was found in the tissues of mice through histological examination and clinical serum biochemistry analyses (Figure S4B–D, Supporting Information), suggesting that tRNA^SUAG^ did not cause significant cytotoxicity in vivo. Meanwhile, the occupancy of each serine codon was not significantly affected by tRNA^SUAG^, indicating that tRNA^SUAG^ did not significantly affect tRNA homeostasis in vivo (Figure S4E, Supporting Information). The abundance of RPFs mapped to 3' UTRs of mRNA with the stop codon UAG was comparable between the two groups of mice, suggesting that the global non‐specific readthrough rate for the natural stop codon UAG induced by tRNA^SUAG^ was also comparable to the physiological readthrough rate induced by near‐cognate tRNAs in mouse genes (Figure 4H). Consistent with the natural stop codon UAG, tRNA^SUAG^ also did not cause significant readthrough at natural stop codons UAA and UGA (Figure 4I).

### 2.5 tRNA^SUAG^ Promotes HBc Degradation by Introducing a Phosphorylatable Serine

**Figure 5:**
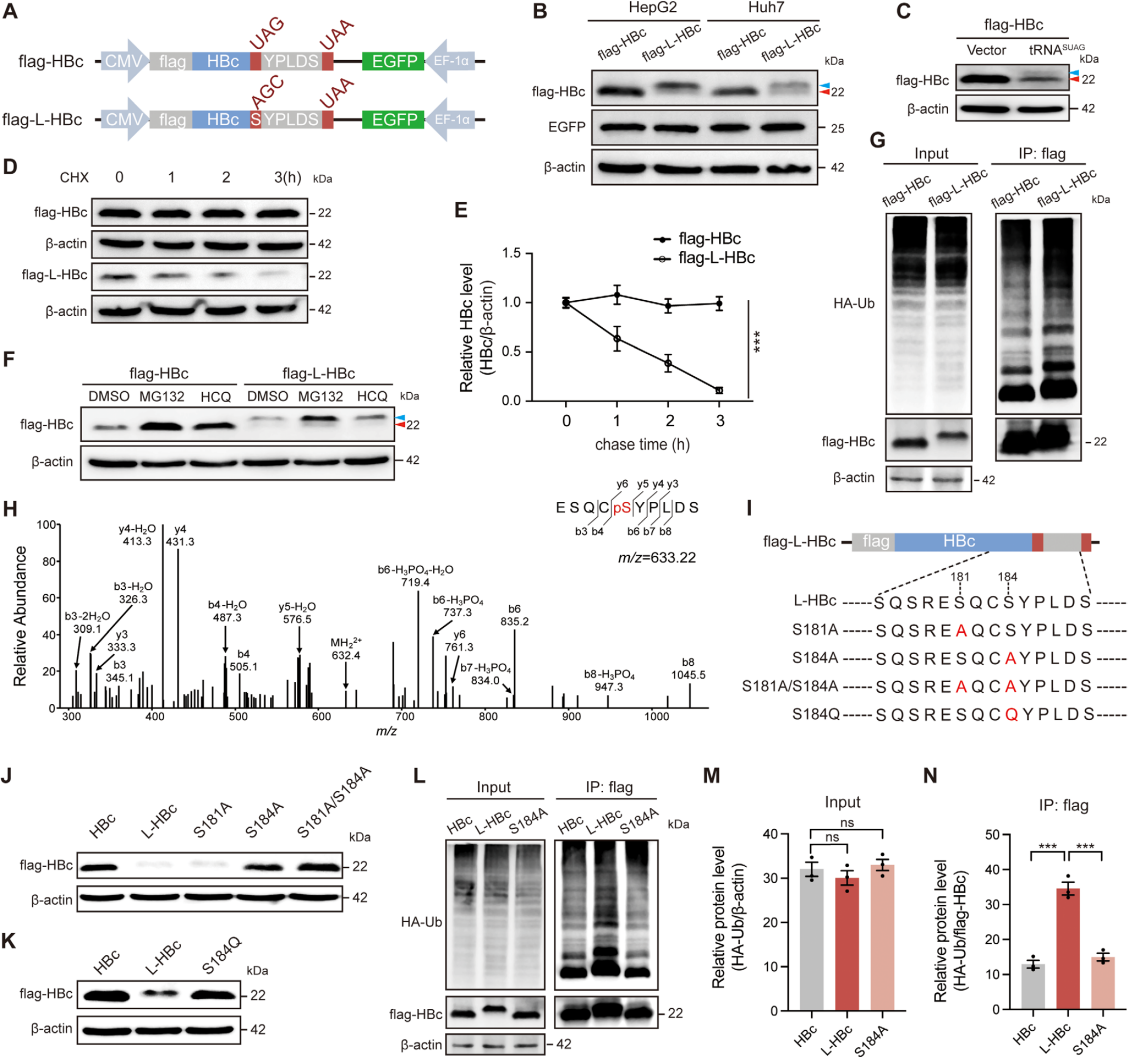
The mechanism of the tRNA^SUAG^‐mediated reduction in HBc levels. A) The diagram of flag‐HBc and flag‐L‐HBc expression cassettes. B) The pCDH‐flag‐HBc or pCDH‐flag‐L‐HBc was transfected into HepG2 and Huh7 cells, and the protein level of HBc was detected by Western blotting. The red arrow indicated the L‐HBc with the same migration rate as wild‐type HBc, and the blue arrow indicated the L‐HBc with a lower migration rate. C) The pCDH‐flag‐HBc and tRNA^SUAG^ expression plasmid or vector control were co‐transfected into HepG2 cells. By increasing the sample loading amount and prolonging the exposure time, the protein level of HBc was detected by Western blotting. D) The pCDH‐flag‐HBc or pCDH‐flag‐L‐HBc was transfected into HepG2 cells, and a cycloheximide (CHX)‐chase assay was performed by adding CHX (50 µg mL^−1^) into the medium at 48 h post‐transfection. The cells were harvested after CHX incubation for 0, 1, 2, and 3 h, and the protein levels of flag‐HBc and flag‐L‐HBc were detected by Western blotting. E) The relative protein levels of flag‐HBc and flag‐L‐HBc were analyzed by ImageJ. F) HepG2 cells were transfected with pCDH‐flag‐HBc or pCDH‐flag‐L‐HBc, and treated with DMSO, MG132 (15 µM), or HCQ (50 µM) for 6 h at 48 h post‐transfection, and the protein level of HBc was detected by Western blotting. G) HepG2 cells were co‐transfected with pCMV‐HA‐Ub and pCDH‐flag‐HBc or pCDH‐flag‐L‐HBc, and treated with MG132 (15 µM) for 6 h at 48 h post‐transfection, and the protein levels of the ubiquitinated flag‐HBc and flag‐L‐HBc were detected by immunoprecipitation (IP) with anti‐flag and Western blotting. H) The phosphorylation of S184 was analyzed by LC‐MS. I) The diagram of the mutated L‐HBc. S: serine; A: alanine; Q: glutamine, and J, K), the corresponding protein levels were detected by Western blotting. L) HepG2 cells were co‐transfected with pCMV‐HA‐Ub and the flag‐tagged HBc, L‐HBc, or S184A mutated L‐HBc expression plasmid. At 48 h post‐transfection, the cells were treated with MG132 (15 µM) for 6 h, and the protein levels of the ubiquitinated flag‐tagged HBc, L‐HBc, and S184A mutated L‐HBc were detected by IP with anti‐flag and Western blotting. The relative protein levels of HA‐Ub in M) Input group and N) IP group were analyzed by ImageJ. ***p < 0.001, ns indicated no significance, two‐tailed *t*‐test.

To explore the mechanism of tRNA^SUAG^‐mediated reduction in HBc levels, a tRNA^SUAG^‐induced longer HBc (L‐HBc) expression plasmid was constructed by mutating UAG into the codon AGC encoding serine (**Figure** **5**A). As shown in Figure 5B, the protein level of L‐HBc was significantly lower than that of wild‐type HBc, which was consistent with the tRNA^SUAG^‐mediated reduction in HBc levels. Notably, we observed two bands of L‐HBc: the migration rate of the weaker band was the same as that of wild‐type HBc, whereas the migration rate of the stronger band was lower than that of wild‐type HBc. Meanwhile, by increasing the loading amount and prolonging the exposure time, we also observed the same two bands of HBc in HepG2 cells co‐transfected with flag‐HBc and tRNA^SUAG^ expression plasmids, which further confirmed that tRNA^SUAG^ could induce the production of L‐HBc by reading through the stop codon of HBc (Figure 5C). As L‐HBc is only six amino acids longer than wild‐type HBc, it is not sufficient to cause significant changes in migration rate, indicating that some post‐translational modifications may lead to a lower migration rate of L‐HBc.

The stability of L‐HBc was significantly lower than that of wild‐type HBc (Figure 5D,E). It has been reported that HBc is degraded through ubiquitin‐proteasome and lysosome‐dependent pathways.^[^ ^25^, ^26^, ^27^ ^]^ To uncover the mechanism of L‐HBc degradation, the pCDH‐flag‐HBc or pCDH‐flag‐L‐HBc plasmid‐transfected HepG2 cells were treated with MG132, a proteasome inhibitor, or hydroxychloroquine (HCQ), a lysosome inhibitor. The results showed that the level of L‐HBc was restored by treating with MG132 but not with HCQ (Figure 5F). Compared to wild‐type HBc, the ubiquitination level of L‐HBc increased (Figure 5G), indicating that L‐HBc was mainly degraded through the ubiquitin‐proteasome pathway. To explore whether L‐HBc affected capsid assembly, the levels of capsids formed by wild‐type HBc and L‐HBc were detected by native agarose gel electrophoresis. The results showed that the relative levels of capsids formed by wild‐type HBc and L‐HBc were comparable, but the size of the capsid formed by L‐HBc was larger than that of wild‐type HBc, which might be due to the fact that L‐HBc was longer than wild‐type HBc (Figure S5A,B, Supporting Information).

It has been reported that the post‐translational modifications of HBc, especially phosphorylation in the C‐terminal domain (CTD) of HBc, can regulate the stability and function of HBc.^[^ ^28^ ^]^ The serine introduced by tRNA^SUAG^ is a phosphorylatable amino acid. Therefore, the phosphorylation status of wild‐type HBc and L‐HBc was analyzed by LC‐MS to further elucidate the mechanism of tRNA^SUAG^‐mediated degradation of HBc. For the known phosphorylation sites of HBc,^[^ ^29^, ^30^ ^]^ the phosphorylation levels were comparable between wild‐type HBc and L‐HBc, whereas the serine introduced by tRNA^SUAG^ (S184) and the adjacent serine (S181) were phosphorylated in L‐HBc (Figure 5H; Figure S6A–D, Supporting Information). To explore whether the phosphorylated S181 and S184 mediated L‐HBc degradation, S181 and S184 were mutated individually or in combination to unphosphorylatable alanine (A), or S184 was mutated to unphosphorylatable glutamine (Q) (Figure 5I). The results showed that the protein level and migration rate of L‐HBc recovered to be comparable to wild‐type HBc when S184 was mutated to A or Q (Figure 5J,K). Moreover, the ubiquitination level of L‐HBc also recovered to be comparable to wild‐type HBc when S184 was mutated to A (Figure 5L–N), suggesting that the serine introduced by tRNA^SUAG^ was a key phosphorylation site contributing to the ubiquitin‐proteasome degradation of HBc.

### 2.6 tRNA^SUAG^‐gHBV Tandem Array Exhibits a Combined Inhibitory Effect on HBV Replication

**Figure 6:**
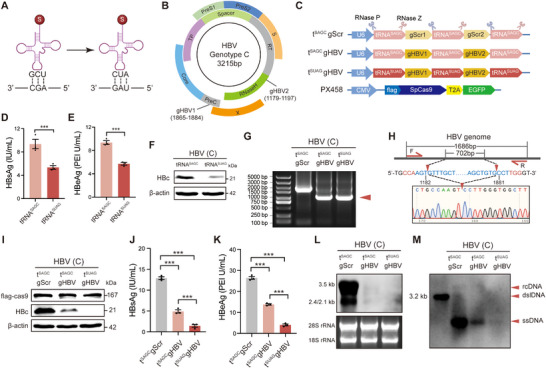
The effect of tRNA^SUAG^‐gHBV tandem array on HBV replication. A) The diagram of tRNA^SUAG^ and its parental tRNA (tRNA^SAGC^). B) The genomic locations targeted by gHBV1 and gHBV2. C) The diagram of the tRNA^SAGC^‐gScr1‐tRNA^SAGC^‐gScr2‐tRNA^SAGC^ (tRNA^SAGC^gRNA^Scr^), tRNA^SAGC^‐gHBV1‐tRNA^SAGC^‐gHBV2‐tRNA^SAGC^ (tRNA^SAGC^gHBV), and tRNA^SUAG^‐gHBV1‐tRNA^SUAG^‐gHBV2‐tRNA^SUAG^ (tRNA^SUAG^gHBV) tandem arrays, as well as the SpCas9‐T2A‐EGFP expression cassette in pSpCas9(BB)‐2A‐GFP (PX458) plasmid. Genotype C 1.3×HBV and tRNA^SAGC^ or tRNA^SUAG^ expression plasmid were co‐transfected into HepG2 cells. At 3 days post‐transfection, the levels of D) HBsAg and E) HBeAg in the culture supernatants were detected by chemiluminescence immunoassays. F) The protein level of HBc was detected by Western blotting. Genotype C 1.3×HBV, PX458, and each tandem array expression plasmid were co‐transfected into HepG2 cells. At 3 days post‐transfection, G) the dual gHBVs/Cas9‐mediated cleavage of HBV genome was detected by PCR, followed by agarose gel electrophoresis, and H) the truncated PCR fragment was sequenced. I) The protein levels of HBc and flag‐cas9 were detected by Western blotting. The levels of J) HBsAg and K) HBeAg in the culture supernatants were detected by chemiluminescence immunoassays. L) The level of intracellular HBV RNA was detected by Northern blotting. M) The level of intracellular HBV DNA was detected by Southern blotting at 5 days post‐transfection. ***p < 0.001, two‐tailed *t*‐test.

To develop a multi‐target therapeutic strategy based on ACE‐tRNA, an (ACE‐tRNA)‐gRNA tandem array was constructed to integrate ACE‐tRNA with CRISPR/Cas9 technology. By combining tRNA^SUAG^ with our group's previously designed HBV‐specific gRNAs (gHBV1 and gHBV2),^[^ ^31^ ^]^ a tRNA^SUAG^‐gHBV1‐tRNA^SUAG^‐gHBV2‐tRNA^SUAG^ tandem array was constructed, in which the parental tRNA of tRNA^SUAG^ (tRNA^SAGC^), scramble gRNA1 (gScr1), and scramble gRNA2 (gScr2) were used as the negative control of tRNA^SUAG^, gHBV1, and gHBV2, respectively (Figure 6A–C). First, we confirmed that tRNA^SUAG^ could also significantly inhibit HBV replication by reducing HBc compared to tRNA^SAGC^ (Figure 6D–F). Further, we found that the DNA sequence between the gHBV1/Cas9 and gHBV2/Cas9 cleavage sites was efficiently removed through tRNA^SAGC^‐gHBV1‐tRNA^SAGC^‐gHBV2‐tRNA^SAGC^ and tRNA^SUAG^‐gHBV1‐tRNA^SUAG^‐gHBV2‐tRNA^SUAG^ tandem arrays, and a shortened HBV genome was formed, suggesting that gHBV1 and gHBV2 could be efficiently released from the two tandem arrays (Figure 6G,H). Compared to tRNA^SAGC^‐gScr1‐tRNA^SAGC^‐gScr2‐tRNA^SAGC^ tandem array, tRNA^SAGC^‐gHBV1‐tRNA^SAGC^‐gHBV2‐tRNA^SAGC^ and tRNA^SUAG^‐gHBV1‐tRNA^SUAG^‐gHBV2‐tRNA^SUAG^ tandem arrays significantly inhibited HBV replication, and the ability of tRNA^SUAG^‐gHBV1‐tRNA^SUAG^‐gHBV2‐tRNA^SUAG^ tandem array in inhibiting HBV replication was significantly higher than that of tRNA^SAGC^‐gHBV1‐tRNA^SAGC^‐gHBV2‐tRNA^SAGC^ tandem array (Figure 6I–M).

**Figure 7:**
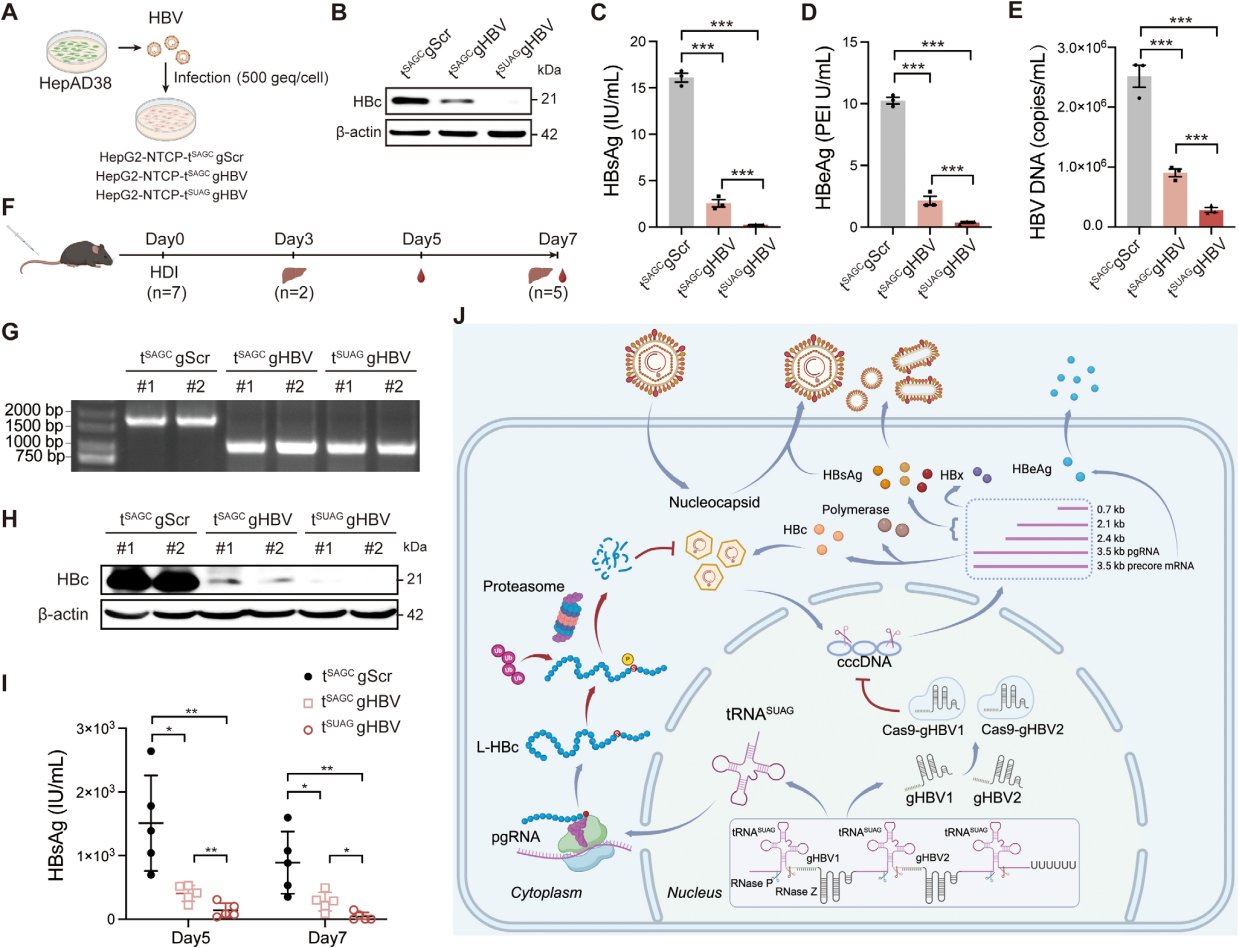
The effect of tRNA^SUAG^‐gHBV tandem array on HBV replication under HBV infection and in vivo. A) The diagram on HBV infection (500 geq/cell). B) The cells were harvested at 7 days postinfection, and the protein level of HBc was detected by Western blotting. The levels of C) HBsAg and D) HBeAg in the culture supernatants were detected by chemiluminescence immunoassays. E) The level of HBV DNA in the culture supernatants was detected by qPCR. F) The workflow of HDI mouse model. G) The dual gHBVs/Cas9‐mediated cleavage of HBV genome in liver tissues was detected by PCR, followed by agarose gel electrophoresis at 3 days postinjection. H) The protein levels of HBc in liver tissues were detected by Western blotting at 3 days postinjection. I) The level of HBsAg in the serum was detected by chemiluminescence immunoassay at 5 days and 7 days post‐injection, respectively. J) The diagram of the dual‐target inhibitory effect on HBV replication mediated by tRNA^SUAG^‐gHBV tandem array (Created with BioRender.com). *p < 0.05, **p < 0.01, ***p < 0.001, two‐tailed *t*‐test.

Next, we established HepG2‐NTCP‐tRNA^SUAG^‐gHBV1‐tRNA^SUAG^‐gHBV2‐tRNA^SUAG^ cells stably expressing tRNA^SUAG^‐gHBV1‐tRNA^SUAG^‐gHBV2‐tRNA^SUAG^ tandem array in HepG2‐NTCP cells, which was confirmed by HBc‐Nluc reporter (Figure S7A, Supporting Information). HepG2‐NTCP‐tRNA^SUAG^‐gHBV1‐tRNA^SUAG^‐gHBV2‐tRNA^SUAG^ and control cells were infected with HBV (500 geq/cell) produced by HepAD38 cells, and the effect of tRNA^SUAG^‐gHBV1‐tRNA^SUAG^‐gHBV2‐tRNA^SUAG^ tandem array on HBV replication was detected at 7 days post‐infection (**Figure** **7**A). The results showed that tRNA^SUAG^‐gHBV1‐tRNA^SUAG^‐gHBV2‐tRNA^SUAG^ tandem array could also achieve a combined inhibitory effect on HBV replication in the HBV‐infected HepG2‐NTCP‐tRNA^SUAG^‐gHBV1‐tRNA^SUAG^‐gHBV2‐tRNA^SUAG^ cells without cytotoxicity (Figure 7B–E; Figure S7B, Supporting Information).

Moreover, the effect of tRNA^SUAG^‐gHBV tandem array on HBV replication was assessed using a hydrodynamic injection (HDI) mouse model (Figure 7F). Consistently, a shortened HBV genome was efficiently formed by tRNA^SAGC^‐gHBV1‐tRNA^SAGC^‐gHBV2‐tRNA^SAGC^ and tRNA^SUAG^‐gHBV1‐tRNA^SUAG^‐gHBV2‐tRNA^SUAG^ tandem arrays, suggesting that gHBV1 and gHBV2 could also be efficiently released from the two tandem arrays in vivo (Figure 7G). Meanwhile, tRNA^SUAG^‐gHBV1‐tRNA^SUAG^‐gHBV2‐tRNA^SUAG^ tandem array could also achieve a dual‐target combined inhibitory effect on HBV replication in vivo without cytotoxicity (Figure 7H,I; S8A–C, Supporting Information).

## 3 Discussion

Given that RNA therapy has been widely explored for treating various diseases, the delivery systems and stability‐enhancing modifications of ACE‐tRNA have received sufficient support.^[^ ^32^, ^33^ ^]^ However, the safety of ACE‐tRNA is still crucial for its clinical applications. Since the structure of 3’ UTR, poly(A)‐binding protein, and sequences including tetra‐nucleotides signal around the natural stop codons contribute to preventing the nonspecific readthrough for the natural stop codons,^[^ ^34^, ^35^, ^36^ ^]^ the nonspecific readthrough rate of natural stop codons induced by ACE‐tRNA is as low as the physiological readthrough rate induced by near‐cognate tRNA.^[^ ^14^, ^15^ ^]^ In this study, we further confirmed through Ribo‐seq and quantitative proteomics analyses that ACE‐tRNA was difficult to read through the normal stop codons of human genes, demonstrating the potential safety of ACE‐tRNA and supporting its future clinical applications. To further ensure the safety of ACE‐tRNA therapy for HBV infection, liver‐specific targeting strategies can be applied, including the use of hepatotropic AAV8 for liver tissue‐specific delivery and the use of liver cell‐specific promoters to control ACE‐tRNA expression. These strategies can significantly improve the targeting specificity toward liver cells, thereby further reducing potential off‐target effects and providing further safety assurances for the therapeutic applications of ACE‐tRNA in HBV infection.

Gene overlap is widespread in viral genomes, allowing the virus to encode more information within a relatively small genome.^[^ ^37^ ^]^ For the HBV genome, the partial overlap between the C ORF and P ORF, as well as another highly conserved in‐frame stop codon behind the stop codon in the C ORF, makes the stop codon of the C ORF like PTC. In this study, we found that the stop codon of HBc is UAG and highly conserved, indicating that the normal termination of HBc translation is crucial for HBV replication. ACE‐tRNAs, including tRNA^SUAG^, tRNA^LUAG^, and tRNA^QUAG^, can read through the stop codon of HBc, which may be due to the similarity between the stop codon of HBc and PTC. Among the three ACE‐tRNAs, only tRNA^SUAG^ could efficiently reduce the level of HBc protein. Notably, tRNA^SUAG^ can also significantly reduce the levels of HBsAg, HBeAg, HBV RNA, and HBV DNA. Although HBeAg is also located in the C ORF, tRNA^SUAG^ should not directly affect HBeAg level as its C‐terminal domain is removed during HBeAg processing. Once the stop codon of HBc was mutated from UAG to UAA, the decrease in HBc protein levels mediated by tRNA^SUAG^ and its inhibitory effect on HBV replication would disappear, which confirmed that tRNA^SUAG^ can reduce HBc protein levels and inhibit HBV replication by reading through the stop codon of HBc. Since the nucleotide immediately following the stop codon UAG of HBc is U and highly conserved in the C ORF, and UAGU was the most prone to be read through by tRNA^SUAG^ compared to UAGA, UAGG, and UAGC, it can also partially explain why ACE‐tRNA can read through the stop codon of HBc. The effect of tRNA^SUAG^ in inhibiting HBV replication disappeared when blocking HBc expression or supplementing HBc expression that was not affected by tRNA^SUAG^, indicating that the tRNA^SUAG^ mediated inhibition of HBV replication was mediated by reducing HBc protein level. Consistent with our results, some CAMs, including GLS4 and Bay41‐419, can significantly reduce the levels of HBsAg and HBeAg by reducing HBc level or affecting capsid formation.^[^ ^38^ ^]^ In addition, colchicine can also significantly reduce the levels of HBsAg, HBeAg, and HBV RNA through selective autophagic degradation of HBc protein, and this effect also disappears when the expression of HBc is blocked.^[^ ^39^ ^]^

Regarding the mechanism by which tRNA^SUAG^ reduced HBc protein levels, we found that tRNA^SUAG^ promoted HBc degradation through the ubiquitin‐proteasome pathway by reading through the stop codon of HBc and subsequently introducing a phosphorylatable serine. Although L‐HBc induced by tRNA^SUAG^ was six amino acids longer than wild‐type HBc, it was not sufficient to cause a marked change in the migration rate of L‐HBc. Since protein phosphorylation can induce a mobility shift in SDS‐polyacrylamide gel electrophoresis (SDS‐PAGE),^[^ ^40^ ^]^ the lower migration rate of L‐HBc should be attributed to the phosphorylatable serine (Ser184) introduced by tRNA^SUAG^. As expected, the migration rate of L‐HBc recovered to the level of wild‐type HBc when Ser184 in L‐HBc was mutated to unphosphorylatable alanine or glutamine. Compared with the overexpression of L‐HBc, tRNA^SUAG^ induced L‐HBc would not be observed unless the loading amount was increased and the exposure time was prolonged, which might be due to the lower stability of L‐HBc and the lower amount of L‐HBc induced by tRNA^SUAG^.

As an initial adaptive immune system against phages and foreign genetic material in prokaryotes, the CRISPR/Cas9 system is not only widely used for treating various genetic diseases,^[^ ^41^, ^42^, ^43^, ^44^ ^]^ but also for eradicating various viral infections, including HBV infection.^[^ ^45^, ^46^, ^47^, ^48^ ^]^ Since RNase P and RNase Z do not require specific sequences to cleave tRNA precursors,^[^ ^20^ ^]^ gHBV1 and gHBV2 can be efficiently released from the tRNA^SUAG^‐gHBV1‐tRNA^SUAG^‐gHBV2‐tRNA^SUAG^ tandem array. Although the CRISPR/Cas9 system can reduce HBV DNA to relatively low levels, it cannot completely destroy the HBV genome, resulting in the production of low‐level HBV RNA. Under these conditions, the tRNA^SUAG^ expressed by the tRNA^SUAG^‐gHBV1‐tRNA^SUAG^‐gHBV2‐tRNA^SUAG^ tandem array reached a relatively high ratio compared to the remaining HBV RNA, indirectly enhancing the ability of tRNA^SUAG^ to reduce HBc levels. In addition, the tandem array simultaneously expresses tRNA^SUAG^ and two HBV‐specific gRNAs, thereby avoiding the need for repeated administrations. Therefore, the combination of ACE‐tRNA and CRISPR/Cas9 technology holds promise for clearing HBV infection.

In summary, this study successfully employed ACE‐tRNA to introduce a phosphorylatable serine into the C‐terminal domain of HBc by reading through the stop codon of HBc, thereby potentially promoting the ubiquitin‐proteasome degradation of HBc and subsequently inhibiting HBV replication. Based on the tRNA^SUAG^‐gHBV1‐tRNA^SUAG^‐gHBV2‐tRNA^SUAG^ tandem array, a combined inhibition of HBV replication was achieved by integrating ACE‐tRNA and CRISPR/Cas9 technology (Figure 7J). This study is the first to use ACE‐tRNA to promote the degradation of viral protein by introducing an amino acid that can be post‐translationally modified. Since different ACE‐tRNAs exhibit varying readthrough efficiencies depending on the protein and even the specific site,^[^ ^49^ ^]^ systematic screening and optimization of ACE‐tRNAs should be carried out in the future to further enhance the applicability and performance of ACE‐tRNAs. ACE‐tRNA undergoes physiological metabolic processes like natural tRNA, making it safer than traditional compound drugs. In addition, ACE‐tRNA is derived from the mature tRNA that bypasses the processing of pre‐tRNA, which ensures that ACE‐tRNA does not undergo unexpected processing or degradation. Since the aminoacylation process does not rely on the anticodon loop of tRNA,^[^ ^50^ ^]^ ACE‐tRNA derived from the natural tRNA by modifying the anticodon loop does not affect the translational machinery. Compared with small interfering RNA (siRNA) and antisense oligonucleotides (ASO), ACE‐tRNA only targets highly conserved stop codons of viral proteins; thus, the impact of viral mutations on ACE‐tRNA is much lower than that of siRNA or ASO, which require complementary pairing with viral RNA sequences. Altogether, ACE‐tRNA is a promising approach for treating HBV infection. The application of ACE‐tRNA to read through the conserved stop codons of viral proteins with overlapping ORFs in the compact viral genome, and subsequently inhibit viral replication by promoting the degradation of viral proteins, provides a new therapeutic approach for viral infections and expands the application scope of ACE‐tRNA. In addition, this study provides a promising therapeutic strategy for clearing viral infections by combining ACE‐tRNA and CRISPR/Cas9 technology.

## 4 Experimental Section

### Cell Lines

HEK293T, HepG2, and Huh7 cells were maintained in Dulbecco's modified Eagle medium (DMEM) supplemented with 10% fetal bovine serum (FBS) (PAN, Germany). The stable HBV‐expressing cell line HepAD38 was kindly provided by Professor Ningshao Xia at Xiamen University, and was cultured with DMEM containing 10% FBS, 400 µg mL^−1^ G418, and 4 µg mL^−1^ Doxycycline. HepG2‐NTCP cells were kindly provided by Professor Kuanhui Xiang at Peking University, and were cultured with DMEM containing 10% FBS and 400 µg mL^−1^ G418. To avoid G418‐induced read‐through, we only added G418 for selection in the first three passages of cell culture, and then proceeded with subsequent culture without G418.

### Plasmids

The pRL‐TK, genotype A, B, and C 1.3×HBV plasmid, pCMV‐HA‐Ub, and pSpCas9(BB)‐2A‐GFP (PX458) plasmid were maintained in our laboratory. The pU6‐tRNA^SUAG^, pU6‐tRNA^LUAG^, pU6‐tRNA^QUAG^, and pU6‐tRNA^SAGC^ plasmids were constructed by ligating the annealed oligonucleotides into pU6 vector. And the cassettes of U6‐ACE‐tRNAs were inserted upstream to the CMV promoter in the pcDNA3.1‐CMV‐EGFP plasmid, making them bicistronic. The PTC‐containing luciferase reporter plasmid, pCDH‐flag‐HBc, pCDH‐flag‐L‐HBc, pCDH‐D‐HBc, pCDH‐D‐L‐HBc, pCDH‐HBc‐UAA, and HBc‐Nluc reporter plasmids were constructed by pEASY‐Uni Seamless Cloning and Assembly Kit (TransGen Biotech, China). To express an internal reference gene, the cassettes of EF1α‐puroR in pCDH‐flag‐HBc and pCDH‐flag‐L‐HBc plasmids were replaced with EF1α‐EGFP. The pCDH‐flag‐L‐HBc (S181A), pCDH‐flag‐L‐HBc (S184A), pCDH‐flag‐L‐HBc (S181A/S184A), pCDH‐flag‐L‐HBc (S184Q), 1.3×HBV G2452A, and 1.3×HBV△HBc plasmids were constructed by the QuikChange site‐directed mutagenesis method (Agilent Technologies, USA). The corresponding oligonucleotides (Table S1, Supporting Information) and primers (Table S2, Supporting Information) were synthesized in Sangon Biotech (Shanghai, China). The sequences of tRNA‐gRNA tandem array, including tRNA^SAGC^‐gScr1‐tRNA^SAGC^‐gScr2‐tRNA^SAGC^, tRNA^SAGC^‐gHBV1‐tRNA^SAGC^‐gHBV2‐tRNA^SAGC^, and tRNA^SUAG^‐gHBV1‐tRNA^SUAG^‐gHBV2‐tRNA^SUAG^ were synthesized and inserted into pU6 and lentiCRISPR v2 plasmids. The corresponding sequences (Table S3, Supporting Information) were synthesized in Genscript (Nanjing, China).

### Transfection and Construction of Stable Cells

The plasmid transfection was performed with Lipofectamine 3000 (Invitrogen, USA). HepAD38‐NC, HepAD38‐tRNA^SUAG^, HepG2‐NTCP‐NC, HepG2‐NTCP‐tRNA^SUAG^, HepG2‐NTCP‐tRNA^SAGC^‐gScr1‐tRNA^SAGC^‐gScr2‐tRNA^SAGC^, HepG2‐NTCP‐tRNA^SAGC^‐gHBV1‐tRNA^SAGC^‐gHBV2‐tRNA^SAGC^, and HepG2‐NTCP‐tRNA^SUAG^‐gHBV1‐tRNA^SUAG^‐gHBV2‐tRNA^SUAG^ cells were constructed by using lentiviral transduction system. Briefly, the lentivirus prepared in HEK293T cells was used to infect HepAD38 and HepG2‐NTCP cells, and then the corresponding stable cells were selected in medium containing 2 µg mL^−1^ puromycin and maintained in medium containing 1 µg mL^−1^ puromycin.

### Luciferase Reporter Assay

Luciferase reporter assays were performed by Dual‐luciferase assay kit (Promega, USA) or Nano‐Glo luciferase assay kit (Promega, USA), following the manufacturer's instructions.

### Western Blot

Briefly, the membranes were blocked with 5% non‐fat milk for 1 h and then incubated with primary antibodies, including mouse anti‐HBc (MBL, Japan), mouse anti‐flag (MBL, Japan), mouse anti‐HA (MBL, Japan), and rabbit anti‐β‐actin (Abclonal, China) overnight at 4 °C. After washing with TBS‐T three times, the membranes were incubated with HRP‐conjugated Goat Anti‐Mouse or Anti‐Rabbit secondary antibodies (Easybio, China) for 1 h and detected by ChemiScope (Clinx, China).

### Detection of HBsAg and HBeAg

The levels of HBsAg and HBeAg were detected by INFINITE Lumi (Tecan, USA), using chemiluminescence immunoassay kits (Autobio Diagnostics, China).

### Quantification of HBV DNA in the Culture Supernatant

For HBV DNA extraction, 200 µL culture supernatant was harvested and first digested with DNase I (TransGen Biotech, China) for 3 h at 37°C, or 200 µL mouse serum was harvested. Then HBV DNA was extracted with EasyPure Viral DNA/RNA Kit (TransGen Biotech, China). Quantitative PCR (qPCR) was performed by Taq Pro Universal SYBR qPCR Master Mix (Vazyme, China). The corresponding primers were listed in Table S4 (Supporting Information).

### Detection of the Cleaved HBV DNA Fragment

The cleaved HBV DNA fragment was detected as previously reported.^[^ ^31^ ^]^ Briefly, the total genomic DNA was extracted, underwent PCR, and separated by agarose gel (1.2%) electrophoresis. The corresponding primers were listed in Table S4 (Supporting Information).

### Quantification of tRNA^SUAG^

Total RNA was extracted using the TRIzol reagent (Thermo Fisher Scientific, USA). RNA samples were demethylated using rtStarTM tRNA Pretreatment Kit (Arraystar Inc, USA), according to the manufacturer's instructions. Reverse transcription was conducted using stem‐loop primer: 5’‐GTCGTATCCAGTGCAGGGTCCGAGGTATTCGCACTGGATACGACCGACGAG‐3’. The level of tRNA^SUAG^ was detected by qPCR using the forward primer 5’‐TGGTTAAGGCGATGGACTCTA‐3’ and reverse primer 5’‐GTCGTATCCAGTGCAGGGT‐3’.

### Northern Blot

Intracellular total RNA was extracted using the TRIzol reagent (Thermo Fisher Scientific, USA). Northern blot was conducted using the DIG Northern Starter Kit (Roche, Switzerland). In summary, the isolated RNA underwent heat denaturation for 10 min at 65 °C, followed by separation on a 1.5% agarose gel containing MOPS and formaldehyde. The RNA was then transferred onto a Nylon membrane (Roche, Switzerland). The membrane was subsequently hybridized with a Dig‐labeled HBV probe and incubated with anti‐digoxigenin‐AP Fab fragments (Roche, Switzerland) for 1 h at room temperature. The HBV probe was synthesized via PCR using the forward primer 5’‐CTAATCATCTCATGTTCA‐3’, the reverse primer 5’‐GGACTGCGAATTTTGGCC‐3’, and digoxigenin‐11‐dUTP. HBV RNA levels were detected using ChemiDoc XRS Imaging System (Bio‐Rad, USA).

### Southern Blot

The DNA samples were subjected to electrophoresis on a 1.2% agarose gel, followed by transfer onto a nylon membrane (Roche, Switzerland). Subsequent hybridization was performed using a digoxin‐labeled DNA probe that spans the complete HBV genome. The probe was generated from the pBB4.5‐1.3×HBV plasmid and labeled with the DIG‐High Prime DNA Labeling and Detection Starter Kit II (Roche, Switzerland). The forward primer was 5’‐TGGAACCTTTGTGGCTCCTC‐3’, and the reverse primer was 5’‐GGGAGACCGCGTAAAGAGAG‐3’.

### HBV Infection

HBV was harvested from HepAD38 cells and concentrated by PEG8000 (Sigma‐Aldrich, USA). HepG2‐NTCP‐NC, HepG2‐NTCP‐tRNA^SUAG^, HepG2‐NTCP‐tRNA^SAGC^‐gScr1‐tRNA^SAGC^‐gScr2‐tRNA^SAGC^, HepG2‐NTCP‐tRNA^SAGC^‐gHBV1‐tRNA^SAGC^‐gHBV2‐tRNA^SAGC^, or HepG2‐NTCP‐tRNA^SUAG^‐gHBV1‐tRNA^SUAG^‐gHBV2‐tRNA^SUAG^ cells were seeded in a 6‐well plate, and then were inoculated with 500 HBV genome equivalents per cell (500 geq/cell) in the presence of 2% PEG8000 and 2% DMSO for 24 h. After infection, the cells were washed with PBS five times, and then maintained in fresh DMEM containing 2% FBS and 2% DMSO.

### Cell Counting Kit‐8 Assay

Cell viability assay was carried out with Cell Counting Kit‐8 (Dojindo, Japan). Briefly, the cells were seeded in a 96‐well plate. After 48 h, 10 µL CCK‐8 solution was added into each cell, and then the optical density 450 (OD450) was detected by SpectraMax Absorbance Reader (Molecular Devices, USA).

### CHX Chase Assay

To examine the stability of HBc, 50 µg mL^−1^ cycloheximide (CHX) was added to the medium. The cells were harvested at 0, 1, 2, and 3 h with CHX incubation, respectively. The protein levels of HBc were detected by Western blotting.

### Ribo‐seq

Ribo‐seq was conducted by Genedenovo Biotechnology Co., Ltd. Briefly, the metagene plot was generated to display the density of ribosome footprints across the transcriptome, with a focus on the region surrounding the normal stop codon UAG. The plot was normalized to allow for comparison between samples. The RRTS was calculated for each transcript by measuring the density of RPFs in the 3’ UTR between the normal stop codon and the first in‐frame downstream 3’ TC relative to CDS. The average codon occupancies were determined by calculating the ribosome densities at each codon, normalized to the average density for CDS of each transcript. The raw data of Ribo‐seq analyses were uploaded to the Sequence Read Archive (SRA) database (PRJNA1252342).

### Proteomics Analysis

4D Fast DIA quantitative proteomics analysis was conducted in PTM BIO, and the differential gene expression analysis was performed. The raw data of proteomics analysis were uploaded to PRoteomics IDEntifications (PRIDE) database (PXD063150).

### Immunoprecipitation

The cells in a 10 cm dish were harvested at 48 h post‐transfection and lysed in cell lysis buffer, and then anti‐flag agarose beads (Sigma‐Aldrich, USA) were added to the lysate and incubated overnight. After centrifugation, the lysate was removed, and the beads were washed with PBS three times. Finally, the beads were eluted with flag peptide elution buffer, and then the eluted solution was moved to a new tube for Western blotting.

### In‐Gel Protein Digestion and LC‐MS Analysis

The samples obtained from immunoprecipitation were applied to SDS‐PAGE electrophoresis. Coomassie blue‐stained gels were then divided into three slices per sample for further in‐gel digestion. Briefly, gel slices were excised into ≈1 mm^3^ cubes and destained with 50% (v/v) acetonitrile (ACN) and 50 mM NH_4_HCO_3_. To disrupt protein disulfide bonds, gel cubes were reduced with 10 mM dithiothreitol (DTT) in 100 mM NH_4_HCO_3_ at 56°C for 30 min and subsequently alkylated with 55 mM iodoacetamide (IAM) in 100 mM NH_4_HCO_3_ at room temperature for 20 min (in complete darkness). After dehydration by ACN, the in‐gel protein was digested with trypsin overnight at 37 °C. Finally, the tryptic peptides were extracted from gel cubes with 50% (v/v) ACN and 5% (v/v) formic acid (FA) before being dried on a SpeedVac vacuum concentrator for further MS/MS analyses. LC‐MS analyses of peptide samples were carried out on a hybrid ion trap‐Orbitrap mass spectrometer (LTQ Orbitrap Velos, Thermo Fisher Scientific, USA) coupled with nanoflow reversed‐phase liquid chromatography (EASY‐nLC 1200, Thermo Fisher Scientific, USA).

### Animal Experiments

For AA8 transduction, AAV8‐1.3×HBV (genotype D) and AAV8‐tRNA^SUAG^ or AAV8‐NC were diluted in 200 µL PBS and were injected into the tail vein of male C57BL/6J mice (5 weeks old). There were seven mice in each group. Three mice in each group were sacrificed at 4 weeks postinjection, and the other four mice in each group were sacrificed at 12 weeks postinjection. Liver tissues were collected from the sacrificed mice and frozen in liquid nitrogen. Serum was collected from the inner canthus of the mouse every two weeks, and the levels of HBsAg and HBeAg in the serum were detected every two weeks. The levels of HBc in the liver tissue and HBV DNA in the serum were detected at 4 weeks and 12 weeks post‐injection. The levels of ALT and AST in the serum were detected at 12 weeks post‐injection. Ribo‐seq for liver tissues of three mice in each group, and HE staining for liver, heart, muscle, and spleen tissues was performed at 12 weeks postinjection.

For hydrodynamic injection (HDI), genotype C 1.3×HBV, PX458, and pU6‐t^SAGC^gScr, pU6‐t^SAGC^gHBV, or pU6‐t^SUAG^gHBV plasmid were hydrodynamically injected into the tail vein of male C57BL/6J mice (5 weeks old) in 2 mL sterile PBS within 5 s. There were seven mice in each group, two mice in each group were sacrificed at 3 days post‐injection, and the other five mice in each group were sacrificed at 7 days post‐injection. Liver tissues were collected from the sacrificed mice and frozen in liquid nitrogen. Serum was collected from the inner canthus of mice, and the levels of HBsAg and HBeAg in serum were detected at 5 days and 7 days postinjection. The level of HBc in the liver tissue was detected at 3 days postinjection. The levels of ALT and AST in the serum were detected at 7 days post‐injection. HE staining for liver tissues was performed at 7 days post‐injection.

Animal experiments were approved by the Ethics Committee of Peking University (PUIRB‐LA2023501) and were carried out according to the guidelines established by the Institutional Animal Care and Use Committee at Peking University Health Science Center.

### Statistical Analysis

GraphPad Prism 9.0 software (GraphPad Software, USA) was used for statistical analysis. The two‐tailed *t*‐test was used for analyzing two groups. *p *< 0.05 was considered statistically significant.

## Conflict of Interest

The authors declare that they do not have any conflict of interest.

## Author Contributions

J.W. designed and supervised the study. X.Y., H.S., Z.L., Q.Z., and Y.S. performed the research. All authors analyzed the data. X.Y. wrote the original draft. J.W., J.L., and X.L. revised the paper. All authors approved the final manuscript.

## Supporting information



Supporting Information

## Data Availability

The data that support the findings of this study are available from the corresponding author upon reasonable request.
