# Supplementary material for: Anticodon Engineered Transfer RNA (tRNASUAG) Inhibits Hepatitis B Virus Replication by Promoting the Degradation of Core Protein
Source: Adv Sci (Weinh). 2025 Sep 12;12(45):e03534. doi: 10.1002/advs.202503534 (PMC12677619; doi:10.1002/advs.202503534)
Supplement: Supplementary file 1 — Supporting Information [file ADVS-12-e03534-s001.docx]

**Anticodon Engineered Transfer RNA (tRNA^SUAG^) Inhibits Hepatitis B Virus Replication by Promoting the Degradation of Core Protein**

Xingwen Yang, Huiying Sun, Ziheng Luo, Qinxin Zhang, Yarong Song, Jie Li, Xiaoyun Liu, Jie Wang

Table of contents

Supplementary figures………………………………………………………………....2

Supplementary tables……………………………………………………….…….…..10

**
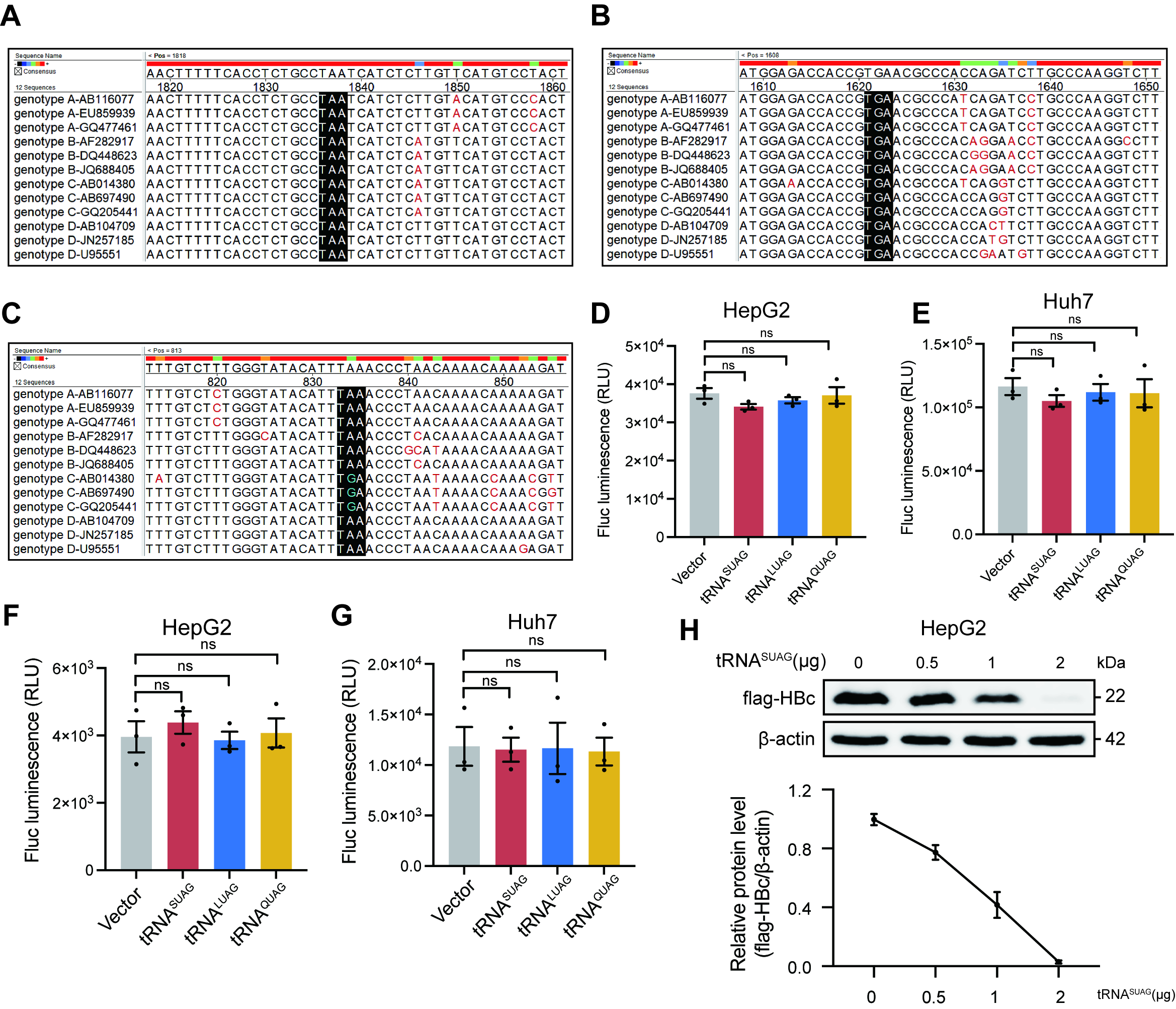
**

# Figure S1. Analyzing the read-through efficiency of ACE-tRNAs targeting UAG. (A) Conservation of the stop codon in the X ORF. (B) Conservation of the stop codon in the P ORF. (C) Conservation of the stop codon in the S ORF. The HBc-Nluc reporter and each ACE-tRNA expression plasmid were co-transfected into (D) HepG2 and (E) Huh7 cells, and the activity of Fluc was detected at 48 h post-transfection. The flag-HBc and each ACE-tRNA expression plasmid were co-transfected into (F) HepG2 and (G) Huh7 cells, and the activity of Fluc was detected at 48 h post-transfection. (H) The flag-HBc expression plasmid and different amount of tRNA^SUAG^ expression plasmid were co-transfected into HepG2 cells, the levels of flag-HBc were detected by Western blotting, and the relative protein levels of flag-HBc were analyzed by ImageJ. ns indicated no significance, two-tailed *t*-test.


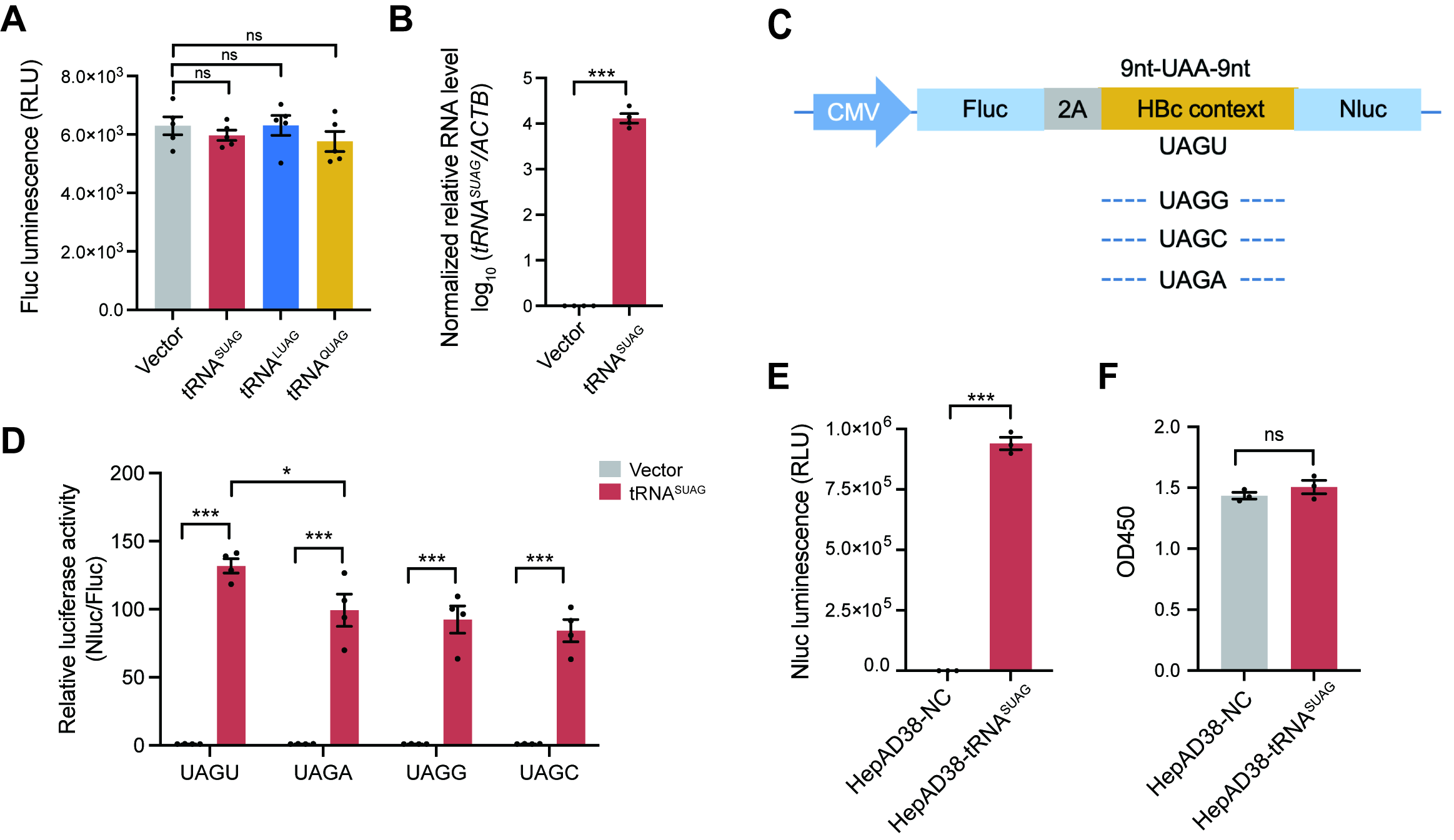


# Figure S2. The effect of ACE-tRNAs on HBV replication. (A) Genotype C 1.3×HBV plasmid and each ACE-tRNA expression plasmid were co-transfected into HepG2 cells, and the activity of Fluc was detected at 48 h post-transfection. (B) Genotype C 1.3×HBV plasmid and tRNA^SUAG^ expression plasmid were co-transfected into HepG2 cells, and the level of tRNA^SUAG^ was analyzed by RT-qPCR. (C) The diagram of Fluc-HBc context Nluc reporters. (D) The Fluc-HBc context Nluc reporters and tRNA^SUAG^ expression plasmid were co-transfected into HepG2 cells, and the relative luciferase activity (Nluc/Fluc) was analyzed. (E) The HBc-Nluc reporter was transfected into HepAD38-NC and HepAD38-tRNA^SUAG^ cells, and the activity of Nluc was detected at 48 h post-transfection. (F) HepAD38-NC and HepAD38-tRNA^SUAG^ cells were seeded into 96-wells plate, and CCK-8 assay was performed after 48 h. **P*<0.05, ****P*<0.001, ns indicated no significance, two-tailed *t*-test.


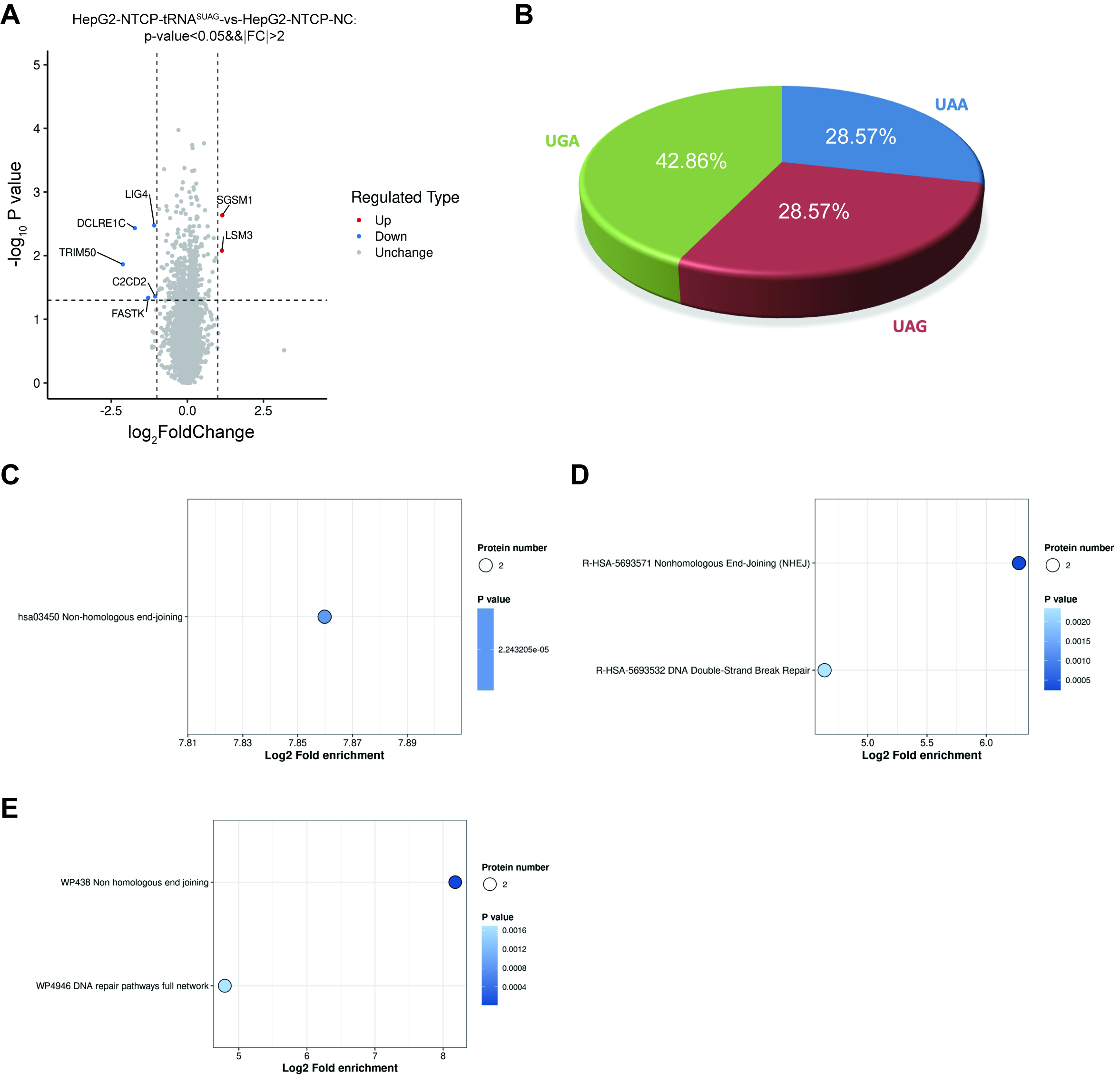


# Figure S3. The quantitative proteomics analysis. (A) Volcano plot showing the differentially expressed proteins between HepG2-NTCP-NC and HepG2-NTCP-tRNA^SUAG^ cells. Red and blue dots denote significantly upregulated and downregulated proteins (*p*<0.05 and fold change>2), respectively. (B) The stop codon distribution of the differentially expressed proteins. Bubble plots of enrichment analysis for differentially expressed proteins, including (C) KEGG pathway analysis, (D) Reactome analysis, and (E) WikiPathways analysis.


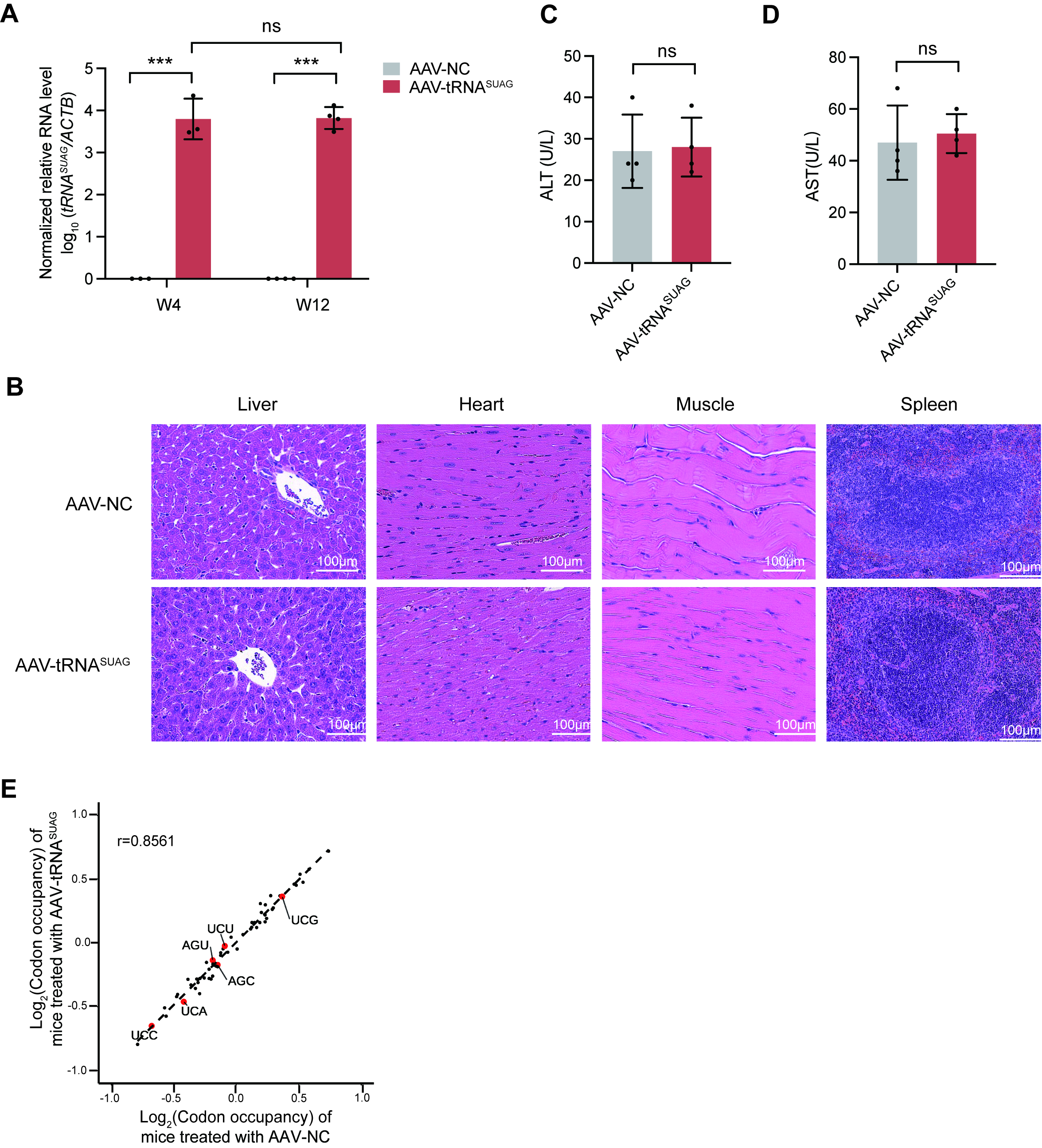


# Figure S4. Analyzing the safety of tRNA^SUAG^ in mice treated with AAV-tRNA^SUAG^. (A) The levels of tRNA^SUAG^ in the liver tissues of mice in each group were analyzed by RT-qPCR. (B) The representative micrographs of the liver, heart, muscle, and spleen tissues stained by hematoxylin and eosin (HE) at 12 weeks post-injection. The levels of (C) ALT and (D) AST in the sera of four mice in each group were detected at 12 weeks post-injection. (E) Scatter plot showed the codon occupancy through exhibiting the RPF densities at each codon in the liver tissues of three mice in each group, and six serine codons were highlighted in red. ****P*<0.001, ns indicated no significance, two-tailed *t*-test.


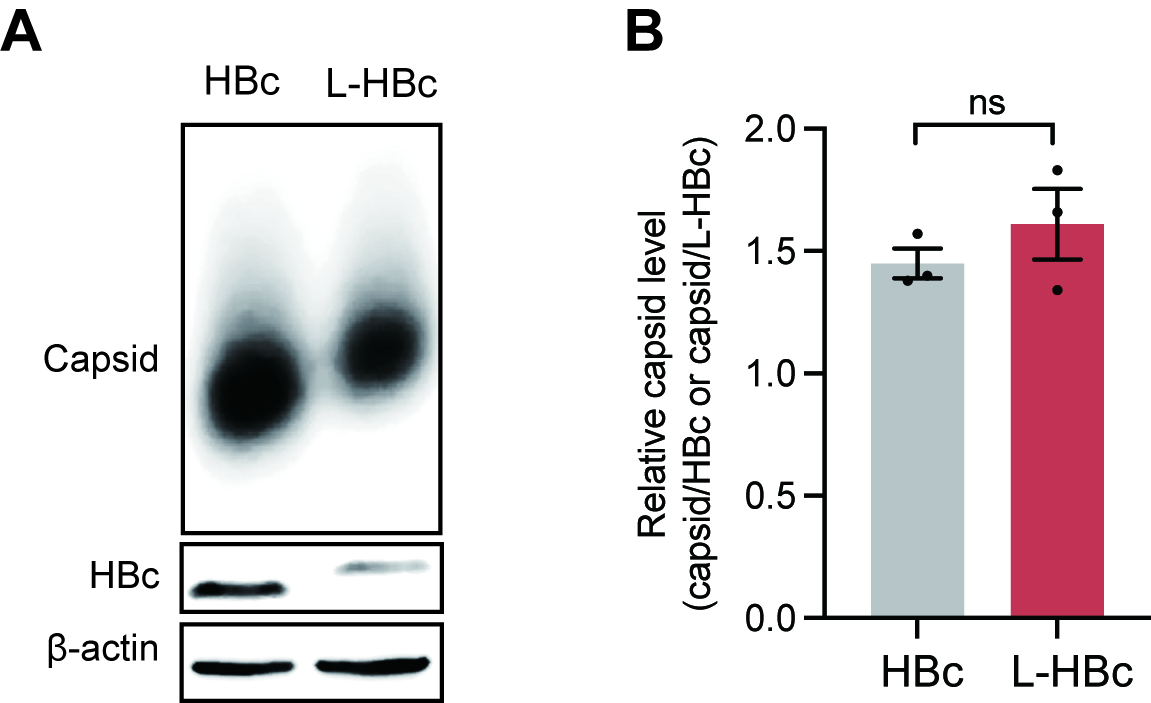


# Figure S5. Detection of capsids formed by HBc and L-HBc. (A) The genotype D HBc (pCDH-D-HBc) or L-HBc (pCDH-D-L-HBc) expression plasmid was transfected into HepG2 cells, and the levels of capsid formed by HBc and L-HBc were detected by native agarose gel electrophoresis. (B) The relative levels of capsids by wild-type HBc (capsid/HBc) and L-HBc (capsid/L-HBc) were quantitated by ImageJ. ns indicated no significance, two-tailed *t*-test.


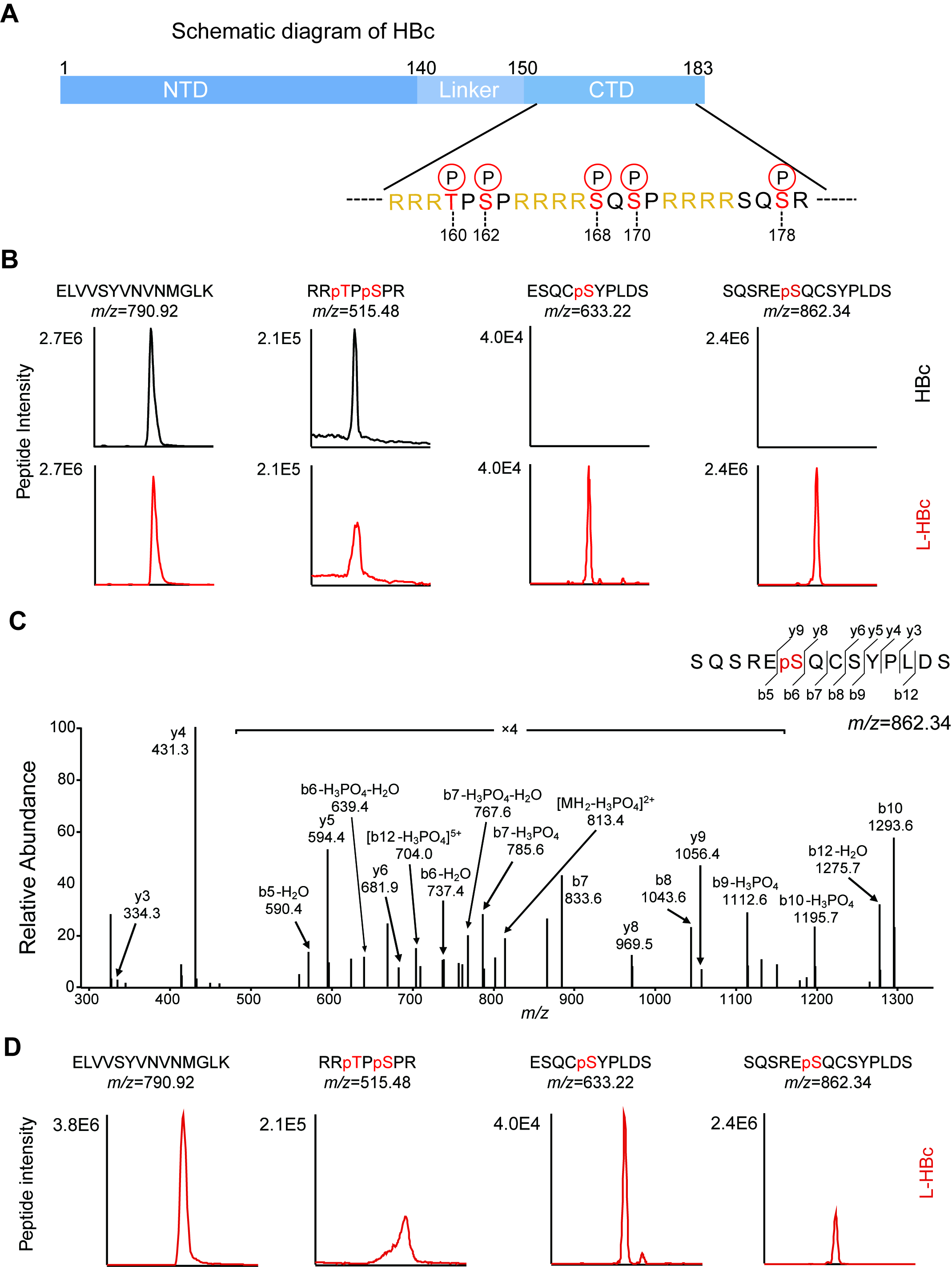


# Figure S6. The phosphorylation of HBc and L-HBc. (A) The diagram of the reported phosphorylation in CTD of HBc. (B) The phosphorylation of HBc and L-HBc in HepG2 cells was detected by LC/MS. The peak intensities indicating the relative amounts of the unmodified peptides (lane 1: aa112-125, as control), and modified peptides (lane 2: aa158-164, as positive control; lane 3: aa180-189; lane 4: aa176-189). (C) LC/MS showed the phosphorylated S181 in the fragmentation of SQSRESQCSYPLDS. (D) The phosphorylation of L-HBc in Huh7 cells was detected by LC/MS. NTD: N terminal domain, CTD: C terminal domain.


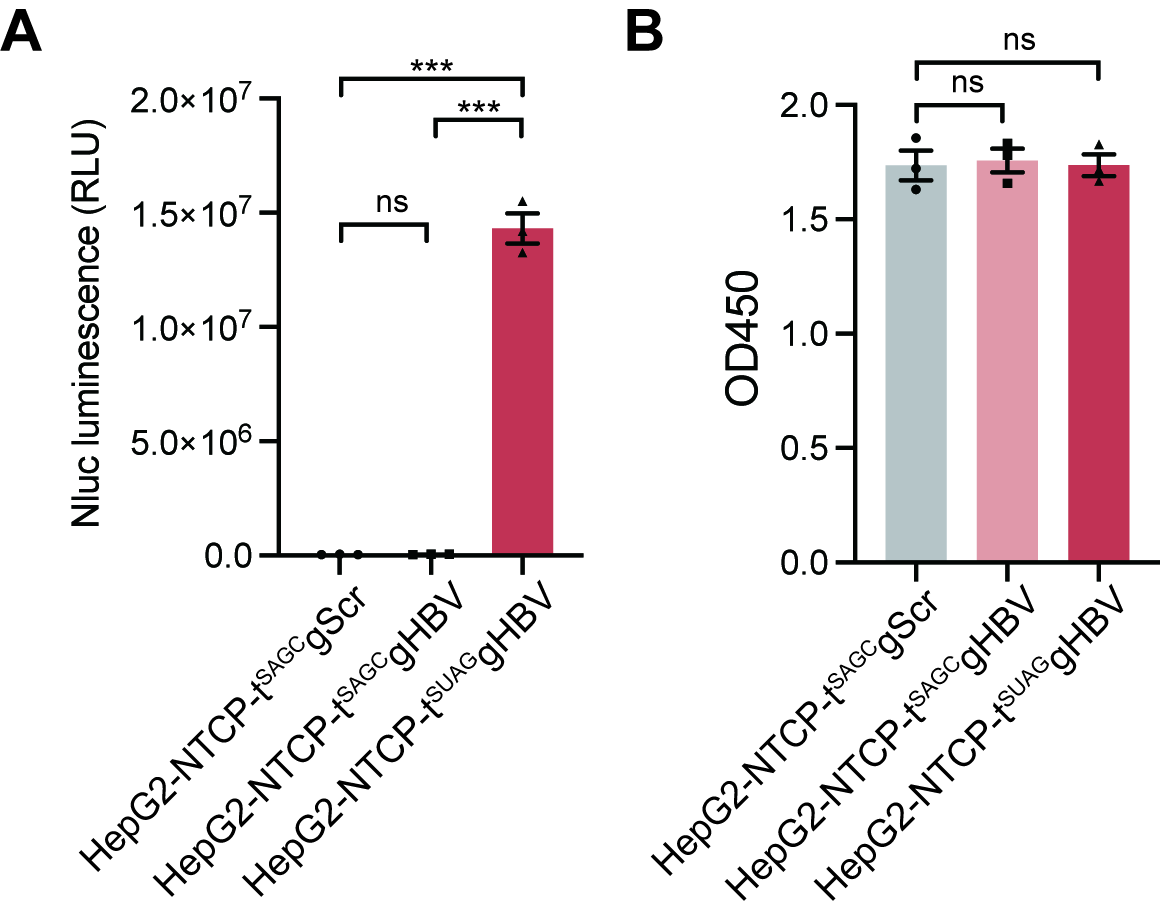


# Figure S7. Analyzing the expression and cytotoxicity of tandem arrays in cells. (A) The HBc-Nluc reporter was transfected into HepG2-NTCP-t^SAGC^gScr, HepG2-NTCP-t^SAGC^gHBV, and HepG2-NTCP-t^SUAG^gHBV cells, and the activity of Nluc was detected at 48 h post-transfection. (B) HepG2-NTCP-t^SAGC^gScr, HepG2-NTCP-t^SAGC^gHBV, and HepG2-NTCP-t^SUAG^gHBV cells were seeded into 96-wells plate, and CCK-8 assays were performed after 48 h. ***indicated *P*<0.001, ns indicated no significance, two-tailed *t*-test.


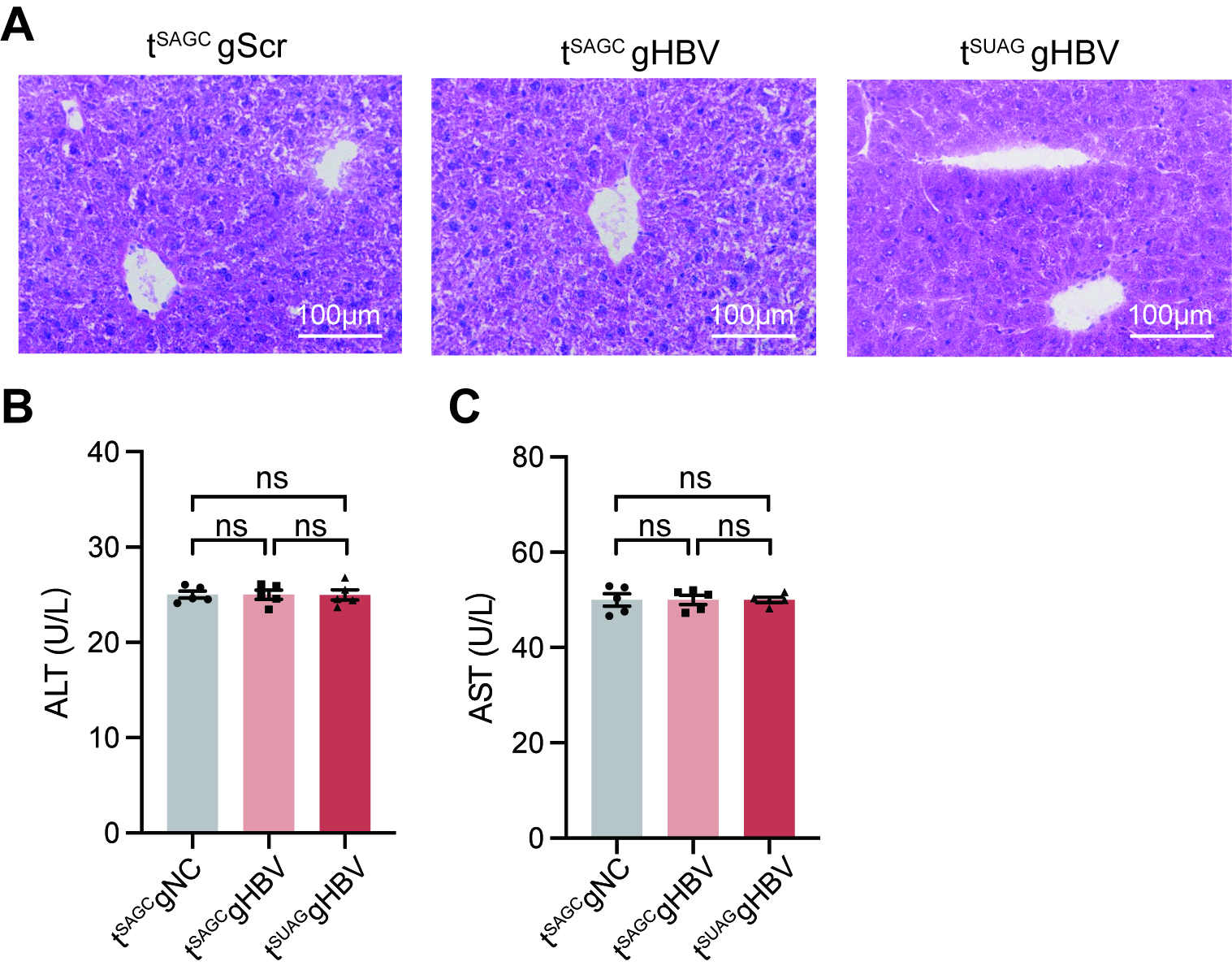


# Figure S8. Analyzing the cytotoxicity of tandem arrays *in vivo*. (A) The representative micrographs of the liver tissues stained by hematoxylin and eosin (HE) at 7 days post-injection. The levels of (B) ALT and (C) AST in the sera of five mice in each group were detected at 7 days post-injection. ns indicated no significance, two-tailed *t*-test.

# Table S1. Sequences of the oligonucleotides used for constructing ACE-tRNAs expression plasmids.

| Name | Oligonucleotides Sequence (5'-3') |
| --- | --- |
| SUAG F | GATCCGACGAGGTGGCCGAGTGGTTAAGGCGATGGACTCTAAATCCATTGTGCTCTGCACACGTGGGTTCGAATCCCATCCTCGTCGTTTTTTA |
| SUAG R | AGCTTAAAAAACGACGAGGATGGGATTCGAACCCACGTGTGCAGAGCACAATGGATTTAGAGTCCATCGCCTTAACCACTCGGCCACCTCGTCG |
| LUAG F | GATCCGGTAGCGTGGCCGAGCGGTCTAAGGCGCTGGATTCTAGCTCCAGTCTCTTCGGGGGCGTGGGTTCAAATCCCACCGCTGCCATTTTTTA |
| LUAG R | AGCTTAAAAAATGGCAGCGGTGGGATTTGAACCCACGCCCCCGAAGAGACTGGAGCTAGAATCCAGCGCCTTAGACCGCTCGGCCACGCTACCG |
| QUAG-F | GATCCGGTTCCATGGTGTAATGGTGAGCACTCTGGACTCTAAATCCAGCGATCCGAGTTCGAGTCTCGGTGGAACCTTTTTTTA |
| QUAG-R | AGCTTAAAAAAAGGTTCCACCGAGACTCGAACTCGGATCGCTGGATTTAGAGTCCAGAGTGCTCACCATTA CACCATGGAACCG |
| SAGC F | GATCCGACGAGGTGGCCGAGTGGTTAAGGCGATGGACTGCTAATCCATTGTGCTCTGCACACGTGGGTTCGAATCCCATCCTCGTCGTTTTTTA |
| SAGC R | AGCTTAAAAAACGACGAGGATGGGATTCGAACCCACGTGTGCAGAGCACAATGGATTAGCAGTCCATCGCC TTAACCACTCGGCCACCTCGTCG |

# Table S2. Sequences of the primers used for plasmid construction.

| Name | Oligonucleotides Sequence (5'-3') |
| --- | --- |
| pCDH-luci-F | GACCTCCATAGAAGATTCTAGGATCCGCCACCATGGAAGACGCCAAAAACATAAAG |
| luci-TGA-R | AGATCCTTCGCGGCCTCTAGATCACACGGCGATCTTTCCGCCCTTC |
| luci-UAG-F | GAGTCCTTCGATAGGGACAAGTAGACAATTGCACTGATCATG |
| luci-UAG-R | CATGATCAGTGCAATTGTCTACTTGTCCCTATCGAAGGACTC |
| flag-HBc-F | CGCGGATCCCTGCAGGCCGCCACCATGGATTACAAGGACGACGATGACAAGGACATTGACCCGTATAAAG |
| HBc-R | CCGGAGCGATCGCAGATCCTTCGCGGCCTCTAGAGATATCTTATGAGTCCAAGGGATAC |
| Nluc-F | GGGAATCTCAATGTTAGTATCCCTTGGACTCAGTCTTCACACTCGAAGATTTCG |
| Nluc-R | AGATCCTTCGCGGCCTCTAGAGATATCTCACGCCAGAATGCGTTCGCAC |
| HBcUAA-R | ATCGCAGATCCTTCGCGGCCTCTAGATTAACATTGAGATTCCCGAGATTG |
| G2452A-F | GATCTCAATCTCGGGAATCTCAATGTTAATATCCCTTGGACTCATAAGGTGGGAAAC |
| G2452A-R | GTTTCCCACCTTATGAGTCCAAGGGATATTAACATTGAGATTCCCGAGATTGAGATC |
| HBV△HBc-F | CGAGATCTCCTCGACACCGCCTCAGCTTAATATCGGGAGGCCTTAGAGTCTCCGG |
| HBV△HBc-R | CCGGAGACTCTAAGGCCTCCCGATATTAAGCTGAGGCGGTGTCGAGGAGATCTCG |
| D-HBc-F | ATAGAAGATTCTAGGATCCCTGCAGGCCGCCACCATGGACATCGACCCTTATAAAG |
| D-HBc-R | GGAGCGATCGCAGATCCTTCGCGGCCTCTAGATTATGAGTCCAAGGAATACTAACATTG |
| D-L-HBc-R | GCGATCGCAGATCCTTCGCGGCCTCTAGATTATGAGTCCAAGGAATACGAACATTGAGG |
| S181A-R | CAGATCCTTCGCGGCCTCTAGAGATATCTTATGAGTCCAAGGGATACGAACATTGAGCTTCCCGAGATTG |
| S184A-R | GATCCTTCGCGGCCTCTAGAGATATCTTATGAGTCCAAGGGATACGCACATTGAGATTC |
| S181A/S184A-R | CAGATCCTTCGCGGCCTCTAGAGATATCTTATGAGTCCAAGGGATACGCACATTGAGCTTCCCGAGATTG |

# Table S3. Sequences of the synthesized tRNA-gRNA tandem arrays.

| Name | Sequence (5'-3') |
| --- | --- |
| tRNA^SAGC^-gScr1-tRNA^SAGC^-gScr2-tRNA^SAGC^ | GGATCCGACGAGGTGGCCGAGTGGTTAAGGCGATGGACTGCTAATCCATTGTGCTCTGCACACGTGGGTTCGAATCCCATCCTCGTCGCACGCATCAACGCCGTTCATGTTTTAGAGCTAGAAATAGCAAGTTAAAATAAGGCTAGTCCGTTATCAACTTGAAAAAGTGGCACCGAGTCGGTGCGACGAGGTGGCCGAGTGGTTAAGGCGATGGACTGCTAATCCATTGTGCTCTGCACACGTGGGTTCGAATCCCATCCTCGTCGGTCAGCATCAACATGCATCGTTTTAGAGCTAGAAATAGCAAGTTAAAATAAGGCTAGTCCGTTATCAACTTGAAAAAGTGGCACCGAGTCGGTGCGACGAGGTGGCCGAGTGGTTAAGGCGATGGACTGCTAATCCATTGTGCTCTGCACACGTGGGTTCGAATCCCATCCTCGTCGTTTTTTTCTAGAGGCCGCGAAGGATCTGCGATAAGCTT |
| tRNA^SAGC^-gHBV1-tRNA^SAGC^-gHBV2-tRNA^SAGC^ | GGATCCGACGAGGTGGCCGAGTGGTTAAGGCGATGGACTGCTAATCCATTGTGCTCTGCACACGTGGGTTCGAATCCCATCCTCGTCGCAAGCCTCCAAGCTGTGCCTGTTTTAGAGCTAGAAATAGCAAGTTAAAATAAGGCTAGTCCGTTATCAACTTGAAAAAGTGGCACCGAGTCGGTGCGACGAGGTGGCCGAGTGGTTAAGGCGATGGACTGCTAATCCATTGTGCTCTGCACACGTGGGTTCGAATCCCATCCTCGTCGGGTTGCGTCAGCAAACACTGTTTTAGAGCTAGAAATAGCAAGTTAAAATAAGGCTAGTCCGTTATCAACTTGAAAAAGTGGCACCGAGTCGGTGCGACGAGGTGGCCGAGTGGTTAAGGCGATGGACTGCTAATCCATTGTGCTCTGCACACGTGGGTTCGAATCCCATCCTCGTCGTTTTTTTCTAGAGGCCGCGAAGGATCTGCGATAAGCTT |
| tRNA^SUAG^-gHBV1-tRNA^SUAG^-gHBV2-tRNA^SUAG^ | GGATCCGACGAGGTGGCCGAGTGGTTAAGGCGATGGACTCTAAATCCATTGTGCTCTGCACACGTGGGTTCGAATCCCATCCTCGTCGCAAGCCTCCAAGCTGTGCCTGTTTTAGAGCTAGAAATAGCAAGTTAAAATAAGGCTAGTCCGTTATCAACTTGAAAAAGTGGCACCGAGTCGGTGCGACGAGGTGGCCGAGTGGTTAAGGCGATGGACTCTAAATCCATTGTGCTCTGCACACGTGGGTTCGAATCCCATCCTCGTCGGGTTGCGTCAGCAAACACTGTTTTAGAGCTAGAAATAGCAAGTTAAAATAAGGCTAGTCCGTTATCAACTTGAAAAAGTGGCACCGAGTCGGTGCGACGAGGTGGCCGAGTGGTTAAGGCGATGGACTCTAAATCCATTGTGCTCTGCACACGTGGGTTCGAATCCCATCCTCGTCGTTTTTTTCTAGAGGCCGCGAAGGATCTGCGATAAGCTT |

# Table S4. Sequences of the primers used for HBV DNA detection.

| Name | Oligonucleotides Sequence (5'-3') |
| --- | --- |
| HBV-DF | CGGCGTTTTATCATMTTCCTCT |
| HBV-DR | GAGGACAAACGGGCAACATAC |
| Cleaved-F | CAACCTCCAATCACTCACCAACC |
| Cleaved-R | GGTGTCGAGGAGATCTCGAATAG |
